# Structural Determinants of Phosphopeptide Binding to the N-Terminal Src Homology 2 Domain of the SHP2 Phosphatase

**DOI:** 10.1021/acs.jcim.0c00307

**Published:** 2020-05-12

**Authors:** Massimiliano Anselmi, Paolo Calligari, Jochen S. Hub, Marco Tartaglia, Gianfranco Bocchinfuso, Lorenzo Stella

**Affiliations:** † Department of Chemical Science and Technologies, University of Rome Tor Vergata, 00133, Rome, Italy; ‡ Theoretical Physics and Center for Biophysics, Saarland University, Campus E2 6, 66123 Saarbrücken, Germany; § Genetics and Rare Diseases Research Division, Ospedale Pediatrico Bambino Gesù, IRCCS, 00146 Rome, Italy

## Abstract

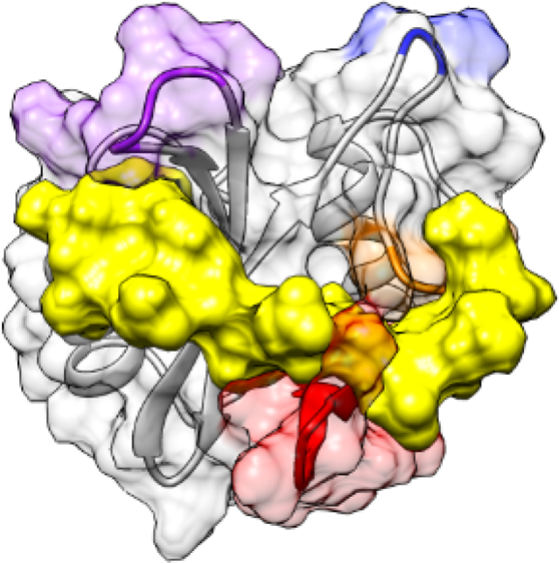

SH2 domain-containing tyrosine phosphatase 2 (SHP2), encoded by *PTPN11*, plays a fundamental role in the modulation of several signaling pathways. Germline and somatic mutations in *PTPN11* are associated with different rare diseases and hematologic malignancies, and recent studies have individuated SHP2 as a central node in oncogenesis and cancer drug resistance. The SHP2 structure includes two Src homology 2 domains (N-SH2 and C-SH2) followed by a catalytic protein tyrosine phosphatase (PTP) domain. Under basal conditions, the N-SH2 domain blocks the active site, inhibiting phosphatase activity. Association of the N-SH2 domain with binding partners containing short amino acid motifs comprising a phosphotyrosine residue (pY) leads to N-SH2/PTP dissociation and SHP2 activation. Considering the relevance of SHP2 in signaling and disease and the central role of the N-SH2 domain in its allosteric regulation mechanism, we performed microsecond-long molecular dynamics (MD) simulations of the N-SH2 domain complexed to 12 different peptides to define the structural and dynamical features determining the binding affinity and specificity of the domain. Phosphopeptide residues at position −2 to +5, with respect to pY, have significant interactions with the SH2 domain. In addition to the strong interaction of the pY residue with its conserved binding pocket, the complex is stabilized hydrophobically by insertion of residues +1, +3, and +5 in an apolar groove of the domain and interaction of residue −2 with both the pY and a protein surface residue. Additional interactions are provided by hydrogen bonds formed by the backbone of residues −1, +1, +2, and +4. Finally, negatively charged residues at positions +2 and +4 are involved in electrostatic interactions with two lysines (Lys89 and Lys91) specific for the SHP2 N-SH2 domain. Interestingly, the MD simulations illustrated a previously undescribed conformational flexibility of the domain, involving the core β sheet and the loop that closes the pY binding pocket.

## Introduction

### SH2 Domains

The idea of protein modularity, with independently folding domains of conserved sequences, began with the discovery of Src homology 2 (SH2) domains.^1^ Their name comes from the identification of sequences of ∼100 amino acids conserved in numerous cytosolic tyrosine kinases, including Src, and the appendix “2” indicates that this module is the second in the Src sequence.^2^ Today, we know that the human genome codes for 121 SH2 domains, contained in 111 distinct proteins.^3,4^ The primary biochemical function of SH2 domains is to selectively recognize polypeptides containing a phosphotyrosine (pY), along with specific contiguous residues.^5^

Tyrosine phosphorylation contributes only ∼0.5% of the total phosphoproteome, yet it plays critical roles in eukaryotic cell regulation.^6^ Substrate specificities of kinases and phosphatases are broad, and their effects in signaling are controlled also by their location. The presence in their structures of domains devoted to protein/protein interactions leads to proper positioning of these enzymes close to their substrates.^7^ In pY signaling, kinases “write” the phosphorylation signal, which can be “erased” by phosphatases. SH2 domains “read” this information, using it to localize signaling proteins correctly.^8^ As a general scheme, binding of an extracellular ligand to a receptor tyrosine kinase induces activation of the receptor, which phosphorylates itself and other nearby proteins. These phosphorylated tyrosine residues then function as docking sites for the SH2 domains of other proteins, which are thus recruited to the cell membrane or activated, causing propagation of the signal.^9^ In addition, SH2 domains enhance tyrosine phosphorylation in vivo by protecting binding sites in their target proteins from dephosphorylation.^10^

### Structure of the SH2 Domains

**Figure 1:**
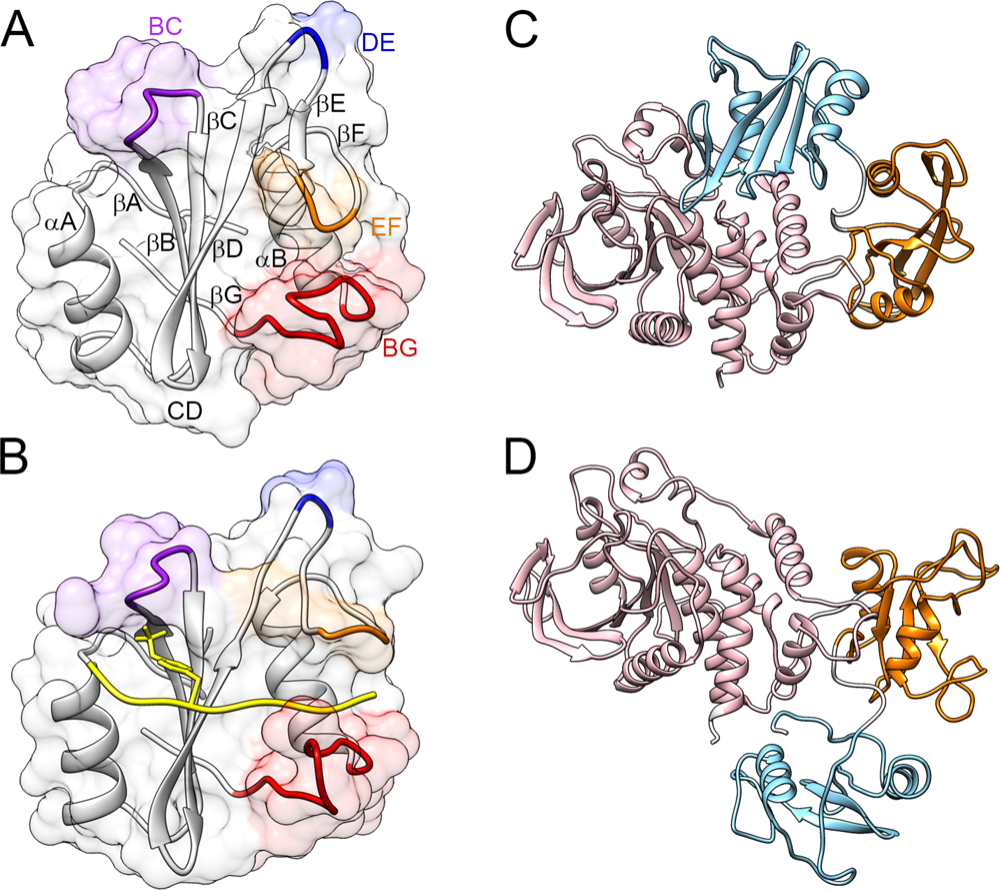
Structure of SHP2: N-SH2 domain and whole protein. (A) The structure of the N-SH2 domain of SHP2 has the βαβββββαβ topology typical of SH2 domains. Loop BC (purple) is part of the pY binding pocket, loop DE (blue) inserts in the PTP active site in the autoinhibited SHP2 conformation, and loops EF (orange) and BG (red) control access to the groove where the phosphopeptide binds. The crystallographic structures of the N-SH2 domain (A) in the autoinhibited conformation of SHP2 and (B) when bound to a phosphopeptide differ mainly for a rearrangement of the EF loop, which in the autoinhibited state blocks the peptide binding site of the N-SH2 domain. SHP2 comprises three domains: N-SH2 (light blue), C-SH2 (orange), and PTP (pink). (C) In the absence of external stimuli, the N-SH2 domain blocks the catalytic site of the PTP domain. (D) Binding of the SH2 domain to phosphorylated sequences or pathogenic mutations favor a conformational transition leading to a rearrangement of the domains and to activation. The SHP2 structures in panels (C) and (D) are reported with their PTP domain in a similar orientation. PDB codes: (A,C) 2SHP, (B) 1AYA, (D) 6CRF.

Three hundred 3D structures of approximately 70 different SH2 domains have been determined. They reveal a highly conserved topology.^6,11^ These domains contain approximately 100 amino acids, with a central β strand, flanked by two α helices. These secondary structure elements are labeled according to their position along the sequence: βA αA βB βC βD βE βF αB βG (Figure 1A). Each residue is then numbered consecutively within the secondary structures.^12^ The central β sheet divides the domain into two functionally distinct sides. The N-terminal side, flanked by helix αA, comprises the conserved pY binding pocket (formed by the BC loop); the C-terminal side, flanked by helix αB and the EF and BG loops, provides a more variable binding surface (specificity determining region) that typically engages residues C-terminal to the pY (Figure 1B).^3,9,13^ The structural arrangement of the domain complexes described above corresponds to the two requirements of SH2 domains: these structural modules (i) must bind only to phosphorylated proteins and (ii) must associate specifically only with certain sequences.

In most structures of SH2-ligand complexes, the phosphopeptide binds across the surface of the domain, orthogonal to the central β sheet, in an extended conformation (Figure 1B),^13^ consistent with the observation that SH2 domains are able to associate with their cognate proteins even when these are denatured.^14^

### SH2-Domain-Containing Protein Tyrosine Phosphatase 2

SH2 domains not only serve to connect the various components of signaling pathways by protein/protein interactions but often also have a role in modulating enzymatic functions. The SH2 domain-containing protein tyrosine phosphatases (PTPs) SHP1 and SHP2 contain two SH2 domains that are N-terminal to the catalytic domain, termed N-SH2 and C-SH2 (Figure 1C). In the absence of external stimuli, the N-SH2 domain interacts with the PTP active site, blocking it.^15^ Association of the SH2 domains with pY motifs favors N-SH2/PTP dissociation and thereby activation of the phosphatase (Figure 1D).^6^ The loss of N-SH2/PTP interactions is triggered by a conformational transition of N-SH2 that leads to a loss of complementarity between the N-SH2 and PTP surfaces.

The SHP2 protein was the first oncogenic PTP discovered. Mutations of *PTPN11* (the gene coding for SHP2) cause more than 30% of cases of juvenile myelomonocytic leukemia (JMML) and are variably found in other childhood malignancies.^16−19^ In addition, SHP2 is required for the survival of receptor tyrosine kinases (RTK)-driven cancer cells,^20^ plays an important role in resistance to targeted cancer drugs,^21^ is a mediator of immune checkpoint pathways,^22^ and is involved in the induction of gastric carcinoma by *Helicobacter pylori*.^23^*PTPN11* mutations also cause the Noonan syndrome and Noonan syndrome with multiple lentigines, two disorders belonging to a family of rare diseases collectively known as RASopathies.^24,25^ For all these reasons, SHP2 is an important molecular target for therapies against cancer and rare diseases.^26−28^ At the molecular level, pathogenic mutations of *PTPN11* often cause an increase in the binding affinity of the SH2 domains of SHP2, leading to hyperactivated signaling of the Ras/MAPK pathway.^29−32^

Due to their role in many signaling pathways, SH2 domains have received much attention as potential targets of pharmaceuticals.^9^ The fact that short pY-containing peptides (usually five to six amino acids) are sufficient to compete with larger protein ligands for SH2 domain binding has prompted researchers both in academia and industry to develop inhibitors of clinically relevant SH2 domains.^33^ However, no molecules targeting the SH2 domains of SHP2 for therapeutic purposes have been reported. Considering its role in the allosteric regulation of SHP2, the N-SH2 domain is particularly interesting under this respect.

### Phosphopeptide Sequence Selectivity of the N-SH2 Domain of SHP2

Several proteins interacting with SHP2 through its SH2 domains have been identified. Lists of more than 50 known or putative interacting proteins have been compiled in the past,^34−36^ and several additional partners have been reported since then.^37−43^ A database of the known interactions is available at phospho.elm.eu.org. However, in many of these cases, the sites of interaction, the pY residues that bind specifically to the SHP2 N-SH2 domain, and the binding affinities have not been determined. Table 1 summarizes the phosphorylated sequences for which a high binding affinity to the N-SH2 domain of SHP2 has been reported. Although exceptions do exist, a general consensus pattern can be clearly detected, with hydrophobic residues (A, L, I, V, M, F, and P) at positions −2, +1, +3, and +5 and acidic amino acids (D or E) at positions 2 and 4.

**Table 1:** Natural Sequences with a High Affinity for the N-SH2 Domain of SHP2^a^

protein	pY	–3	–2	–1	0	+1	+2	+3	+4	+5	+6	relative *K*_d_	ref
Gab1	627	Q	**V**	*E̅*	pY	**L**	*D̅*	**L**	*D̅*	**L**	*D̅*	0.1*	(44)
IRS-1	1179 (1172)	G	**L**	N	pY	**I**	*D̅*	**L**	*D̅*	**L**	**V**	1	(45)
Gab2	614	S	**V**	*D̅*	pY	**L**	**A**	**L**	*D̅*	**F**	Q	2	(46)
IRS-1	896 (895)	**P**	G	*E̅*	pY	**V**	N	**I**	*E̅*	**F**	G	4–8	(47, 48)
SHPS-1	470	T	**L**	T	pY	**A**	*D̅*	**L**	*D̅*	**M**	**V**	10	(49)
CagA		*E̅*	**P**	**I**	pY	**A**	T	**I**	*D̅*	**F**	*D̅*	10	(23)
IRS-1	551 (546)	**I**	*E̅*	*E̅*	pY	T	*E̅*	**M**	**M**	**P**	**A**	10	(47)
PDGFR	1009	S	**V**	**L**	pY	T	**A**	**V**	Q	**P**	N	10–20	(47, 48)
PDGFR	763	*D̅*	**V**	K	pY	**A**	*D̅*	**I**	*E̅*	S	S	n.a.	(50)
SHPS-1	429	*D̅*	**I**	T	pY	**A**	*D̅*	**L**	N	**L**	**P**	n.a.	(51)

a CagA: *H. pylori* virulence factor CagA (cytotoxin-associated gene A); Gab1 and Gab2: GRB2-associated binding proteins 1 and 2; IRS-1: insulin receptor substrate 1; PDGFR: platelet-derived growth factor receptor; SHPS-1: Src homology 2 (SH2)-domain-containing protein tyrosine phosphatase substrate 1 or signal regulatory protein α (SIRPα). Hydrophobic and anionic residues are reported in underlined bold and in overlined italics, respectively. pY numbers refer to the human sequence, except for *H. pylori* CagA, where the sequence refers to the EPIYA-D segment.^23^ For IRS-1 pYs, rat sequence numbers are indicated in parentheses, too, as dissociation constants (*K*_*d*_) were reported for the rat peptides. Relative *K*_*d*_ values are normalized to that of IRS-1 pY1172 (rat sequence, corresponding to human pY1179), i.e., 14 ± 8 nM.^45^ The asterisk indicates that the dissociation constant of Gab1 was measured on a construct containing both the N-SH2 and C-SH2 domains, and the exact phosphopeptide sequence used in the binding assay is unclear due to inconsistencies in the reference.^44^

The sequence selectivity of the N-SH2 domain of SHP2 has been analyzed also by utilizing phosphopeptide libraries. Oriented peptide library studies have examined positions from −1 to +6 with respect to pY. More recently, high-throughput studies with surface-immobilized peptide arrays^31,32,52,53^ analyzed positions from −6 to +6, but distinct preferences were observed only in the −3 to +5 sequence stretch. The results of these investigations are summarized in Table 2. Collectively, a distinct preference for hydrophobic residues at positions −2, +1, +3, and + 5 emerges (consistent with the natural sequences listed in Table 1), while other positions appear to be more variable. In particular, only peptide arrays indicated a possible preference for anionic residues in positions +2 and +4.

**Table 2:**
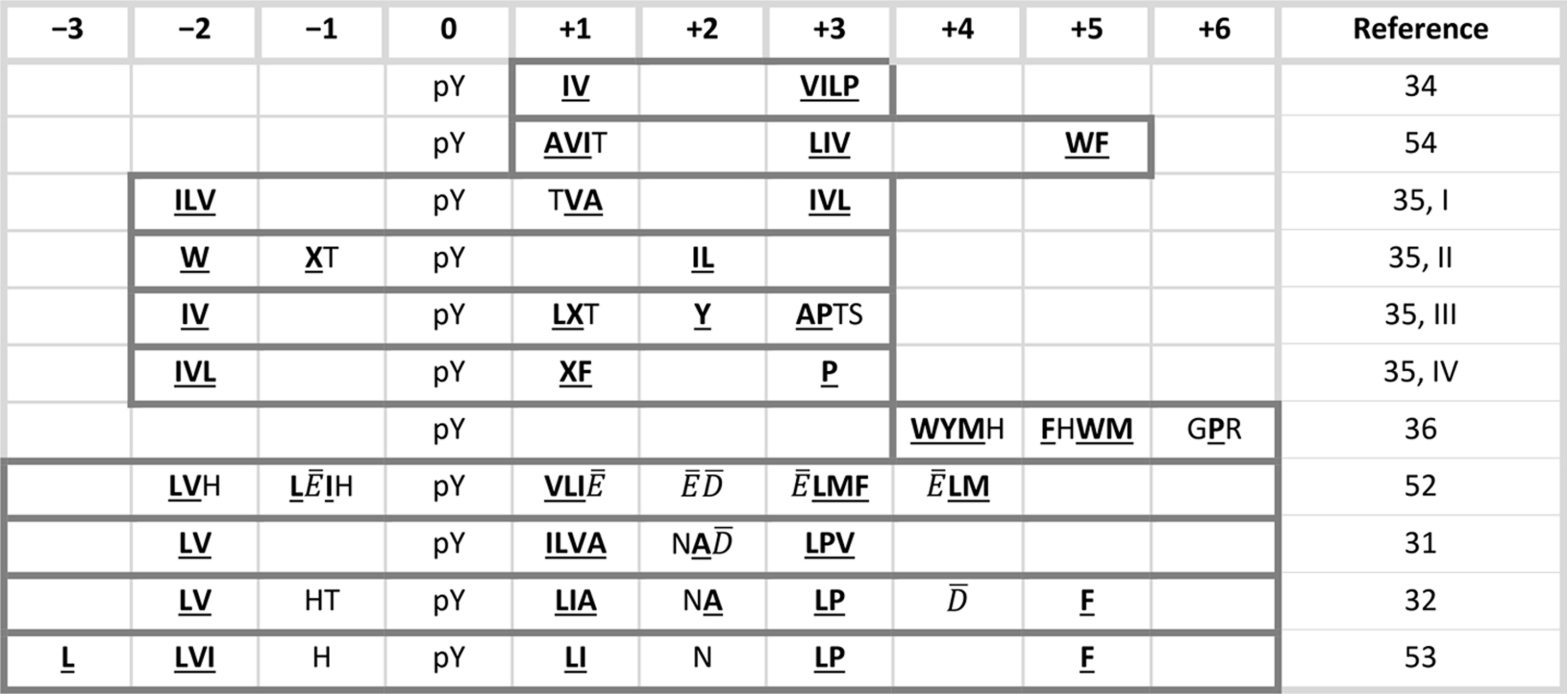
Motifs Determined from Peptide Library Studies^a^

a X = norleucine. The sequence positions investigated in each study have a thicker border. Hydrophobic and anionic residues are reported in underlined bold and in overlined italics, respectively. Roman numerals indicate different peptide classes identified in ref (35).

Distinct selectivity features emerge from these data. Defining the determinants of N-SH2 selectivity is essential to allow the design of new peptides, peptidomimetics, and small molecules targeted to this domain. To this end, we analyzed collectively the available X-ray structures and performed several molecular dynamics (MD) simulations of N-SH2/phosphopeptide complexes.

### Structures of N-SH2/phosphopeptide Complexes and MD Simulations

Seven experimental structures of N-SH2/phosphopeptide complexes, obtained by X-ray crystallography, are available (PDB codes, 3TKZ, 3TL0, 4QSY, 1AYA, 1AYB, 1AYC, 5DF6, 5X7B, and 5X94). In this work, 3TKZ and 1AYC were excluded from further analysis as, in 3TKZ, a non-canonical 1:2 protein/peptide complex is formed,^55^ while in 1AYC the N-SH2 domain is complexed with a nonspecific peptide.^56^ The phosphopeptides present in the remaining structures are listed in Table 3, which include the natural sequences of IRS-1 pY896 (pY895 in rat sequence numbering) (1AYB), PDGFR pY1009 (1AYA), CagA (5X94 and 5X7B), and Gab1 pY627 (4QSY).

**Table 3:** N-SH2/Peptide Complexes (Experimental and Simulated)^a^

method	ID.chain	–7	–6	–5	–4	–3	–2	–1	0	+1	+2	+3	+4	+5	+6	+7	+8	relative *K*_d_	ref
PDB	4QSY.B (*Gab1*)		g	*d̅*	K	Q	**V**	*E̅*	pY	**L**	*D̅*	**L**	*D̅*	**L**	*D̅*			0.1	(44)
	1AYB.P (*IRS-1 895*)				s	**p**	G	*E̅*	pY	**V**	N	**I**	*E̅*	**F**	g	s		4–8	(47), (48)
	1AYA.P (*PDGFR 1009*)					S	**V**	**L**	pY	T	**A**	**V**	Q	**P**	n	*e̅*		10	(47)
	5X94.L (*CagA EPIYA-D*)		**a**	s	**p**	*e̅*	**P**	**I**	pY	**A**	T	**I**	*D̅*	**F**	*D̅*			10	(23)
	3TL0.B (*artificial*)					r	**L**	N	pY	**A**	Q	**L**	**W**	h	r			20	(55)
	5DF6.B (*TXNIP*)	k	**f**	**m**	**p**	**p**	**p**	T	pY	T	*E̅*	**V**	*D̅*					400	(57)
	5X7B.L (*CagA EPIYA-C*)		**v**	s	**p**	*e̅*	**P**	**I**	pY	**A**	T	**I**	*D̅*	*d̅*	**l**			1500	(23)
MD	GAB1_10					Q	**V**	*E̅*	pY	**L**	*D̅*	**L**	*D̅*	**L**	*D̅*			*	
	GAB1_13		G	*D̅*	K	Q	**V**	*E̅*	pY	**L**	*D̅*	**L**	*D̅*	**L**	*D̅*			0.1	(44)
	IRS1-1172_8						**L**	N	pY	**I**	*D̅*	**L**	*D̅*	**L**				*	
	IRS1-1172_9						**L**	N	pY	**I**	*D̅*	**L**	*D̅*	**L**	**V**			*	
	IRS1-1172_11					S	**L**	N	pY	**I**	*D̅*	**L**	*D̅*	**L**	**V**	K		1.0	(45)
	IRS1-1172_12					S	**L**	N	pY	**I**	*D̅*	**L**	*D̅*	**L**	**V**	K	*D̅*	*	
	IRS1-895				S	**P**	G	*E̅*	pY	**V**	N	**I**	*E̅*	**F**	G	S		4–8	(47, 48)
	IMHOF9 (*artificial*)				**A**	**A**	**L**	N	pY	**A**	Q	**L**	**M**	**F**	**P**			5	(36)
	SWEENEY12 (*artificial*)						**V**	**L**	pY	**M**	Q	**P**	**L**	N	G	R	K	8	(35)
	IRS1-546					**I**	*E̅*	*E̅*	pY	T	*E̅*	**M**	**M**	**P**	**A**	**A**		10	(47)
	PDGFR-1009					S	**V**	**L**	pY	T	**A**	**V**	Q	**P**	N	*E̅*		10−20	(47, 48)
	IMHOF5 (*artificial*)					R	**L**	N	pY	**A**	Q	**L**	**W**	H	R			20	(36)

a Hydrophobic and anionic residues are reported in underlined bold and in overlined italics, respectively. Residues in lowercase were not resolved in the crystallographic structures. References indicated in the last column concern data on relative dissociation constant (*K*_d_) values, which were normalized to that of IRS-1 pY1172. IDs of the different simulations will be used, for the sake of brevity, in the rest of the article. Artificial peptide sequences are indicated. Asterisks indicate that the *K*_d_ for the Gab1 peptide was measured with the tandem N-SH2 and C-SH2 domains, and the exact phosphopeptide sequence used in the binding assay is unclear due to inconsistencies in the reference,^44^ and that the Kd for IRS-1 pY1172 refers to the sequence spanning from −3 to +7.^45^

While these structures provide insights into the determinants of N-SH2 selectivity, characterization of the dynamics of domain/peptide complexes is essential to evaluate (i) the stability of the interactions observed in the crystallographic data and (ii) possible conformational transitions of the peptide or of the domain. In addition, no structures are available for the IRS-1 pY1179 peptide (which has one of the highest affinities among known sequences) or for high-affinity artificial peptides that were isolated in library screening studies. To address these issues, we performed 12 (microsecond-long) MD simulations of complexes of the N-SH2 domain with Gab1, IRS-1 pY1172, pY895, pY546 (rat sequence numbering, corresponding to human pY1179, pY896, and pY551), PDGFR pY1009, and three artificial peptides isolated in refs (35) and (36). Moreover, for Gab1 and IRS-1 pY1172, we simulated several analogues of different lengths to check for possible interactions involving N-terminal or C-terminal residues, distant from the pY (Table 3).

## Methods

Initial atomic coordinates were taken from crystallographic structures. As shown in Table S1, for five of the simulated sequences (GAB1_10, GAB1_13, IRS1-895, PDGFR-1009, and IMHOF5), X-ray structures were available, but some residues had to be removed or added. In the other cases (IRS1-1172, IMHOF9, SWEENEY12, and IRS1-546), the sequence to be simulated was obtained by substituting (and adding or removing) some residues, starting from the crystallographic structures listed in Table S1. The termini of the peptides were capped by acetyl and amide groups. These modifications in the peptide molecules were performed by means of Sequence Editor and Protein Builder functionalities in Molecular Operative Environment (MOE) (Chemical Computing Group, Inc.). The backbone of the added residues (at the termini) was initially modeled in an extended conformation. The side chains of the substituted residues were modeled by means of conformational search using a rotamer library as starting guess and allowing repacking. The structures were minimized, with the AMBER12:EHT force field^57^ in generalized Born implicit water,^58^ first on substituted side chains, constraining the backbone, and then on all substituted/added amino acids and on adjacent residues, without restraints, yielding a reasonable binding pose for all peptides. In all cases, the N-SH2 domain comprised residues 3 to 103. Each protein molecule was put at the center of a dodecahedron box, large enough to contain the domain and at least 0.9 nm of solvent on all sides. The protein was solvated with explicit TIP3P water molecules.^59^ All MD simulations were performed with the GROMACS 4.6.5 software package^60^ using the AMBER99SB force field^61^ augmented with the parm99 data set for phosphotyrosine.^62^ Long-range electrostatic interactions were calculated with the particle-mesh Ewald (PME) approach.^63^ A cutoff of 1.2 nm was applied to the direct-space Coulomb and Lennard-Jones interactions. Bond lengths and angles of water molecules were constrained with the SETTLE algorithm,^64^ and all other bonds were constrained with LINCS.^65^ The pressure was set to 1 bar using the weak-coupling barostat.^66^ Temperature was fixed at 300 K using velocity rescaling with a stochastic term.^67^ For all systems, the solvent was relaxed by energy minimization followed by 100 ps of MD at 300 K while restraining protein and peptide atomic positions with a harmonic potential. The systems were then minimized without restraints and slowly equilibrated to remove any possible strains in the starting structures. Their temperature was increased in steps of 50 K from 50 to 300 K. Each step from 50 to 200 K comprised a first stage of 0.5 ns at fixed temperature and a linear temperature ramp of 50 K, lasting 0.5 ns; for the steps from 200 K to 300 K, the duration of these two stages was increased to 1 ns, and then 3 ns were performed at 300 K, for equilibration. Finally, productive runs of 1 μs were performed.

Analysis of structural properties was performed using the GROMACS 2016 analysis tools, on the last 500 ns of the simulations, where convergence of the structural properties was confirmed by block averaging. For crystallographic structures, hydrogen bonds were detected following the usual geometric criteria.^68^ The order parameter Θ_χ_ for the side-chain dihedral angle χ was calculated as
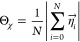
1where the summation is over the *N* frames in the MD trajectory and  is a two-dimensional unit vector whose phase is equal to the dihedral angle χ in structure *i*.^69^ Θ_χ_ = 1 and Θ_χ_ = 0 correspond to a fixed dihedral and free rotation, respectively. In the present work, we limited our analysis only to the order parameter for side-chain dihedral angle χ_1_.

Molecular graphics were prepared with UCSF Chimera (www.cgl.ucsf.edu).

## Results and Discussion

### The −2 to +5 Phosphopeptide Region Interacts Tightly with the Domain

**Figure 2:**
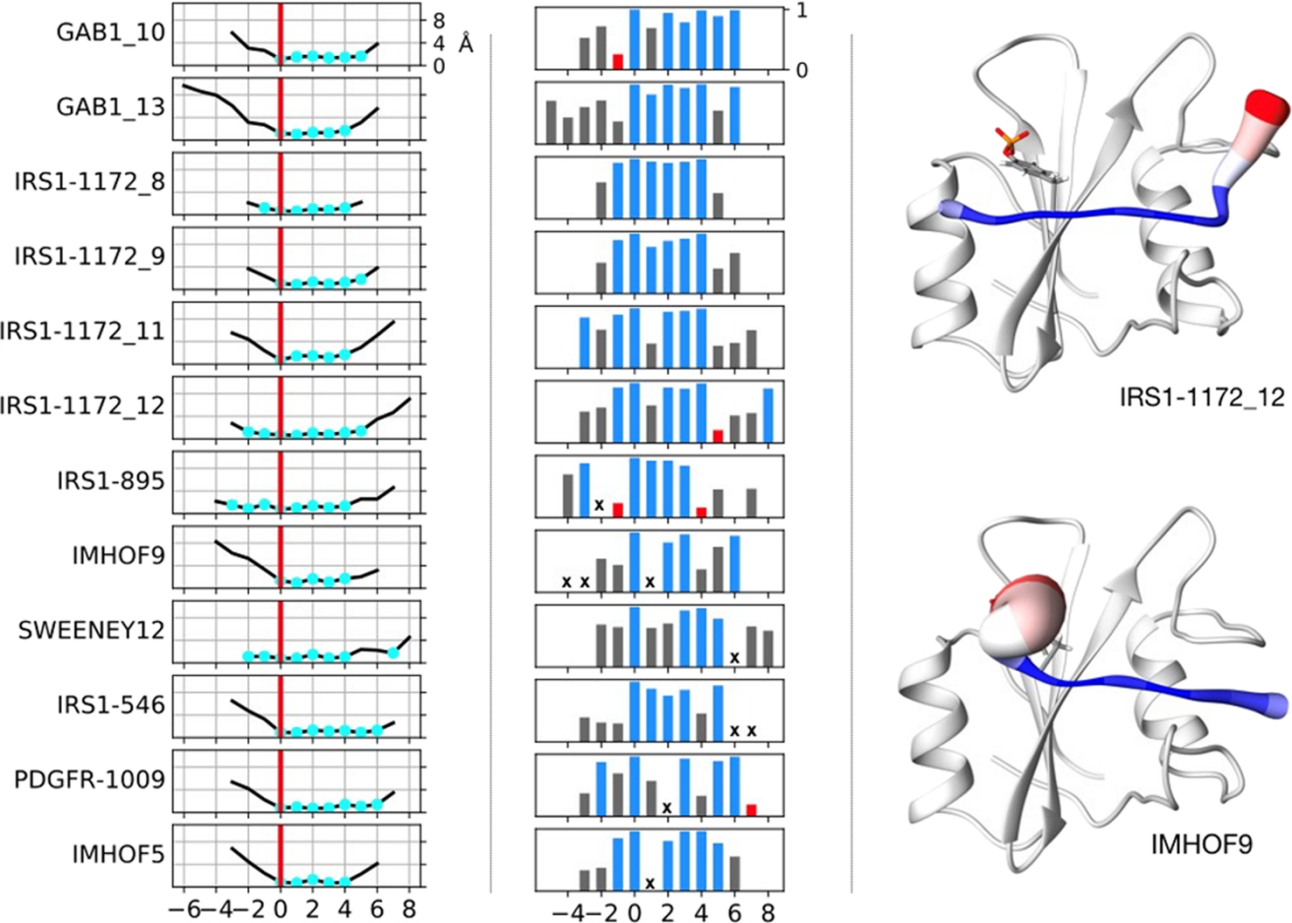
Dynamics of bound peptides. Left panel: RMSF of peptides bound to N-SH2. Residues whose RMSF is less than 1 Å larger than the minimal value are colored in cyan. Middle panel: side-chain order parameter Θ. Values close to unity indicate very narrow dihedral angle distributions and therefore bonds that are rigid with respect to rotation. Bars are colored according to the following scheme: Θ lower than 0.25 (red), between 0.25 and 0.75 (gray), and greater than 0.75 (blue). A bold “x” indicates residues for which the side-chain order parameter cannot be defined (glycines and alanines). Right panel: most representative structures of the IRS1-1172_12 and IMHOF9 simulations, with the peptide backbone size and color (from blue to red) assigned based on the mobility of each residue.

During all simulations, peptides remained in the binding cleft for the whole length of the trajectory. Figure 2 reports the root-mean-square fluctuations (RMSF) of the position of phosphopeptide atoms and the order parameters of the side-chain Cα–Cβ bonds, calculated during the 12 MD simulations. In all cases, RMSF values were less than 1.8 Å for residues in the 0 to +4 interval, indicating a very low mobility for these peptide stretches. Consistently, order parameters were generally higher than 0.75 in this peptide region, although some exceptions were present at positions +1 and +4. In principle, order parameters could be influenced by the size of the side chain, but the fact that we consistently observed high values in the central region of the peptide, irrespective of the peptide sequence, confirms the low mobility of this stretch. In many cases, also, residues −2, −1, and +5 were rather stable, although a larger variability was observed compared to the central stretch. The structures in Figure 2 show that the peptide termini (out of the −2 to +5 region) can detach from the protein. Overall, these findings explain why a distinct selectivity was observed in the peptide library studies only for amino acids falling in the interval from −2 to +5 (Tables 1 and 2). This conclusion is supported by the fact that residues preceding −2 or following +5 are often unresolved in X-ray structures (Table 3).

### The Central Region of the Peptide Is in an Extended Conformation

**Figure 3:**
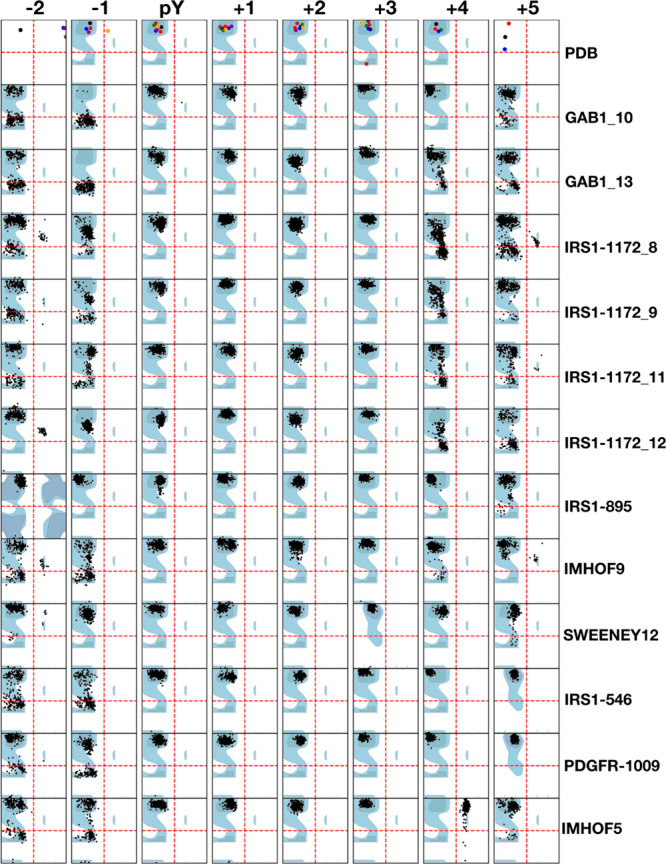
Backbone conformation of the bound peptide residues in PDB X-ray structures and in the simulations. Ramachandran plots of residues from positions −2 to +5 with respect to pY are shown. Crystallographic structures are reported in the first line (“PDB”), with the following color code: 1AYA: green, 1AYB: red, 3TL0: purple, 4QSY: black, 5DF6: orange, 5X7B: brown, 5X94: blue. The allowed regions of the Ramachandran plot are reported in cyan in the background. Angles ϕ and ψ are reported on the *x* and *y* axes, respectively, with values from −180 to +180°. The background shows the allowed regions for a standard amino acid or for Pro or Gly where present (adapted from ref (71)).

Figure 3 shows the Ramachandran plots of the peptide backbone in the X-ray structures and in the MD simulations for residues −2 to +5. In all cases, the dihedral angles of the conformations populated by residues from 0 to +3 fall in the top-left region of the plot, indicating an extremely stable extended structure.^70^ Residues −1 and +4 are extended, too, in all crystallographic structures, but they are more mobile in the simulations, populating regions of the Ramachandran plot corresponding to helical conformations in some cases. Beyond the −1 to +4 region, the backbone conformation is variable.

**Figure 4:**
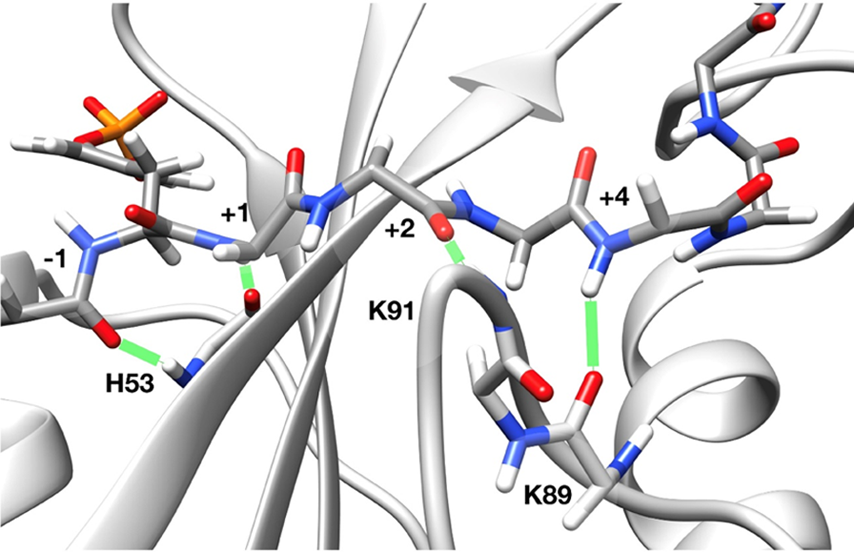
Main H-bonds between the peptide and protein backbones. Most representative structure of the IRS1-1172_12 simulation. H-bonds are highlighted by green lines.

The extended peptide backbone conformation is stabilized by several H-bonds between the peptide and protein backbones, involving peptide residues −1, +1, +2, and +4 and protein residues H53 (βD4), K91 (BG7), and K89 (BG5), as illustrated in Figure 4. These interactions are present in some of the X-ray structures, and they are stably conserved in most of the MD simulations (Table 4). In addition, the MD trajectories show some transient interactions also for the backbone of residue +3 with K91 (BG7) and of +6 with Q86 (BG2) or G87 (BG3), which were not observed in the crystallographic structures.

**Table 4:** Hydrogen Bonds between the Peptide Backbone and the N-SH2 Domain^a^

		–2	–1		+1	+2	+3	+4		+6
method	ID	N	N V51^O^ (βD2)	O H53^N^ (βD4)	N H53^O^ (βD4)	O K91^N^ (BG7)	O K91^Nζ^ (BG7)	N K89^O^ (BG5)	O K89^N^ (BG5)	N Q87^O^ (BG3)
PDB (Å)	4QSY	-	-	-	-	**3.0**	-	**3.0**	-	-
	1AYB	**V51^O^: 2.8**	-	**2.9**	**3.0**	-	-	**3.0**	-	n.a.
	1AYA	-	-	-	**2.7**	**2.8**	-	**2.9**	-	n.a.
	5X94	-	-	-	-	-	-	-	-	-
	3TL0	-	-	**2.9**	**2.9**	**2.5**	-	**2.8**	-	n.a.
	5DF6	-	-	-	**3.0**	**3.2**	-	**2.9**	**2.9**	n.a.
	5X7B	-	-	**3.0**	**2.8**	-	-	**2.9**	-	n.a.
MD (%)	GAB1_10	-	-	-	**73**	17	-	**83**	**90**	**66**
	GAB1_13	-	-	**68**	**96**	**92**	43	**97**	18	18
	IRS1-1172_8	-	-	-	**80**	**74**	36	**90**	-	n.a.
	IRS1-1172_9	-	-	**62**	**88**	**84**	20	**99**	30	26
	IRS1-1172_11	-	-	-	**93**	**82**	29	**94**	-	-
	IRS1-1172_12	-	-	**81**	**91**	**85**	**56**	**99**	-	-
	IRS1-895	**E17^Oε^: 91**	**63**	**93**	**61**	**98**	-	**98**	**91**	**62**
	IMHOF9	-	-	24	**96**	**84**	-	**93**	31	-
	SWEENEY12	-	-	**80**	**91**	**79**	-	**91**	-	-
	IRS1-546	-	-	-	**77**	**97**	-	**96**	**96**	**85**
	PDGFR-1009	-	-	-	**53**	**92**	-	**97**	**96**	**G86^O^: 81**
	IMHOF5	-	-	-	**53**	**69**	-	**99**	-	-

a Stable H-bonds (distance ≤ 3.5 Å in X-ray structures or persistence ≥ 50% in MD simulations) are highlighted in bold. Peptide residues are numbered with respect to the pY position. Backbone atoms involved in hydrogen bonds are shown as apices. Interatomic distances (in Å) are reported for X-ray structures, while % persistence values along the trajectory are shown for MD simulations (see the Methods section). Dashes indicate that the H-bond is not formed in X-ray structures and that it is present for less than 5% in MD simulations. No data are reported for H-bonds that were not stable in at least one of the simulations or structures. Secondary-structure-based residue numbering follows ref (56).

### Phosphotyrosine Interactions

The peptide position in the N-SH2 domain is strongly stabilized also by the interactions of the pY residue with its binding pocket. Several pY interactions are widely conserved in SH2 domains.

The most conserved residue is R βB5 (present in 98% of SH2 domains),^4^ which forms a salt bridge with the phosphate.^72^ This is by far the most stabilizing interaction^73^ and is responsible for the specificity for binding pY (as opposed to other phosphoamino acids): only the lengthy tyrosine side chain allows the phosphate to interact productively with this arginine, whereas serine and threonine are too short.^5,13^

R αA2 (present in 82% of SH2 domains)^4^ interacts with the phosphate group and makes an amino-aromatic interaction with the phenol ring of the pY.^9^

K βD6 is located on the other side of the pY phenol ring from R αA2 so that the two residues together form a clamp around the pY.^9^

The pY recognition site also contains an extensive network of hydrogen bonds.^72^ In particular, S βB7 (present in 88% of SH2 domains) and T/S BC2 form direct hydrogen bonds with the phosphate. The BC loop backbone also contributes to H-bonding.^9^

With respect to these general features of SH2 domains, the N-SH2 domain of SHP2 presents several peculiarities:^72,73^ it has a G in place of R αA2, and in the crystallographic structures, K βD6 contacts the phenol ring solely with its hydrocarbon chain and not with the amine.

Table 5 reports the H-bonds and the salt bridges formed by the phosphate in the crystallographic structures and in the simulations. The general picture described above is confirmed by our analysis of X-ray data. The R βB5 (R32)-pY phosphate ion pair is formed essentially in all structures, while K βD6 (K55) is at a larger distance. H-bonds with S βB7 (S34), S BC2 (S36), and the K BC1 (K35) backbone are consistently formed. An additional H-bond, present in all N-SH2 structures but not conserved in other SH2 domains, is formed with the side chain of T βC3 (T42).

**Table 5:** Hydrogen Bonds and Salt Bridges between pY and N-SH2 Residues*^a^*

		hydrogen bonds					salt bridges		
		S34 (βB7)	K35 (BC1)	S36 (BC2)		T42 (βC3)	R32 (βB5)	K35 (BC1)	K55 (βD6)
method	ID	side-chain Oγ	backbone N	backbone N	side-chain Oγ	side-chain Oγ1			
PDB (Å)	4QSY	**2.7**	**2.6**	**3.0**	**2.7**	**2.8**	4.5	n.a.	5.5
	1AYB	**2.8**	**3.0**	**2.9**	**2.7**	**2.9**	**4.0**	n.a.	4.5
	1AYA	**2.5**	**2.8**	**2.9**	**2.4**	**2.5**	**4.0**	7.9	4.8
	5X94	**3.2**	**3.2**	-	**2.5**	**3.4**	4.5	9.0	6.1
	3TL0	**2.8**	**2.8**	**3.5**	**2.9**	**2.9**	4.2	n.a.	4.7
	5DF6	**3.0**	**3.2**	**2.7**	**2.9**	**2.7**	4.3	n.a.	6.9
	5X7B	-	**2.8**	**2.6**	**3.3**	**3.2**	**4.0**	6.6	n.a.
MD (%)	GAB1_10	**100**	**95**	-	-	**99**	**100**	**79**	-
	GAB1_13	**98**	**94**	-	27	**99**	**100**	**62**	**65**
	IRS1-1172_8	-	-	-	-	**91**	**99**	-	**77**
	IRS1-1172_9	-	-	-	-	38	32	-	**94**
	IRS1-1172_11	**100**	**91**	**85**	**87**	**98**	**80**	-	**50**
	IRS1-1172_12	-	-	-	-	**96**	**99**	-	**82**
	IRS1-895	-	-	-	-	**84**	**91**	-	**75**
	IMHOF9	-	-	-	-	**61**	**83**	-	**69**
	SWEENEY12	20	-	-	-	**84**	**100**	-	**79**
	IRS1-546	**98**	**94**	**51**	**55**	**92**	**99**	36	11
	PDGFR-1009	-	-	-	-	-	**83**	-	**90**
	IMHOF5	-	-	-	-	-	**66**	-	**91**

a Stable bonds (distance ≤ 3.5 Å for H-bonds and ≤ 4.0 Å for salt bridges in X-ray structures or persistence ≥ 50% in MD simulations) are highlighted in bold. Interatomic distances (in Å) are reported for X-ray structures, while % persistence values along the trajectory are shown for MD simulations. Values lower than 5% are omitted. n.a. indicates X-ray structures where lysines 35 or 55 were not resolved in the electron density. Dashes indicate that the bond is not formed in X-ray structures and that it is present for less than 5% in MD simulations. Secondary-structure-based residue numbering follows ref (56).

In the MD simulations, the R βB5 (R32)-pY ion pair is stably maintained, as well as the H-bond formed by T βC3 (T42) (peculiar of SHP2 N-SH2). The other H-bonds are less stable, indicating a significant mobility of the SH2 BC loop.

**Figure 5:**
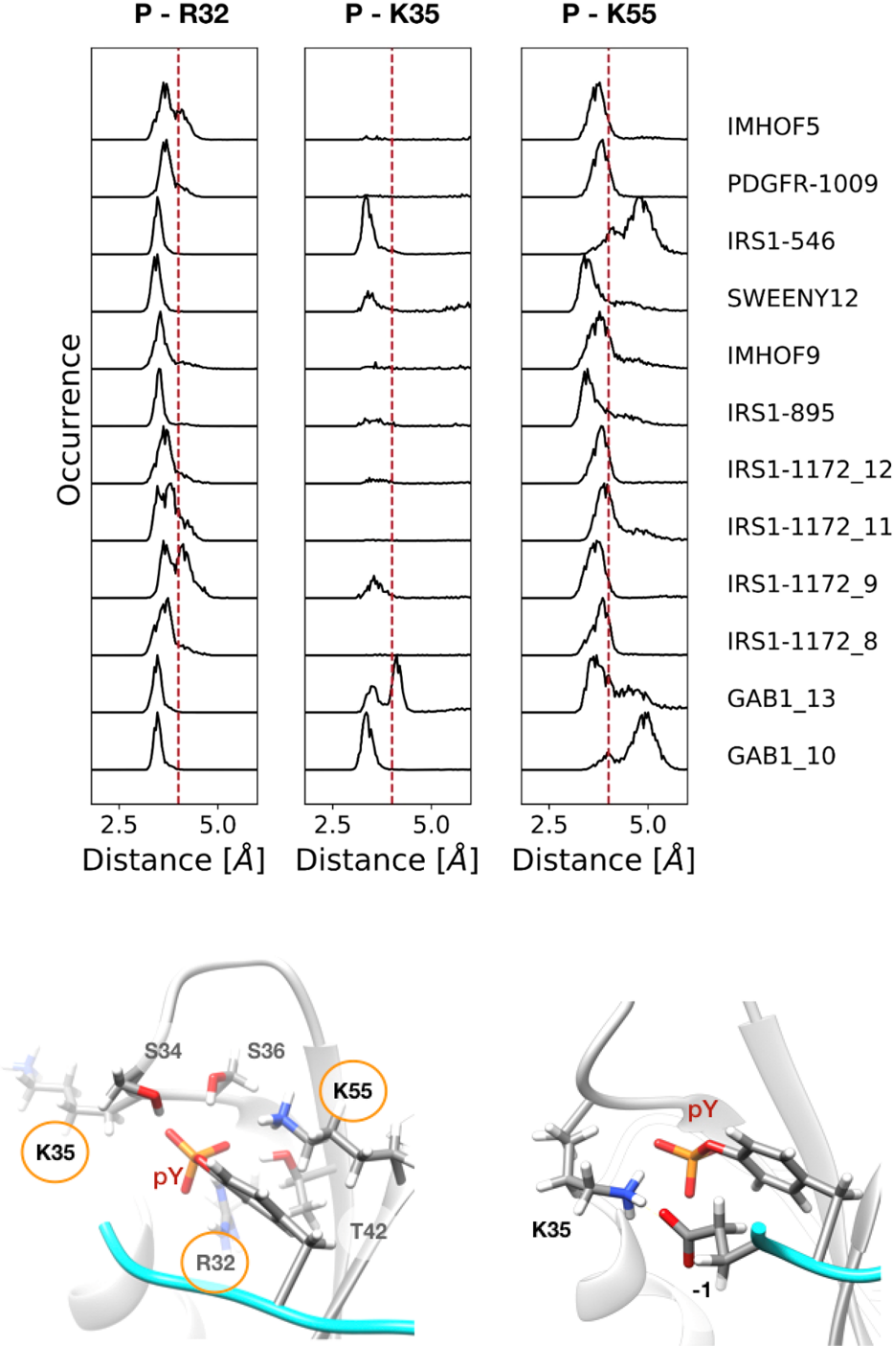
Most common ion-pair interactions between the pY phosphate and N-SH2 residues in MD trajectories. Top panel: distribution of distances between the phosphotyrosine phosphate and protein residues. Distances of less than 4 Å (vertical red dashed lines) are indicative of a stable salt bridge. Bottom panels: N-SH2 residues that interact with the phosphate group of pY (see Table 5) are shown on the left in the most representative structure of the IRS1-1172_8 simulation; the structure on the right shows the alternative arrangement of K35, where it interacts with the pY and a phosphopepeptide anionic residue in −1 (most representative structure of the GAB1_10 simulation).

The distances between the pY phosphate and the charged side chains of R32, K35, and K55 are reported in Figure 5. Interestingly, the possible ion pair with K βD6 (K55), which is conserved in other SH2 domains but is surprisingly not present in crystallographic structures of the N-SH2 domain,^56^ does form often during the simulations. In addition, while the N-SH2 domain lacks the conserved R αA2, it has a K residue in position BC1 (K35), adjacent to the phosphate-binding site. In the crystallographic structures, its side chains point toward the solvent, but in some of the simulations, conformational fluctuations of the BC loop allow the formation of this additional ion pair.

Overall, the MD data suggest that a significant mobility of the pY pocket might be possible while maintaining a strong and stable interaction of the pY residue with the protein domain; when H-bonds are lost, ion pairs can form and vice versa.

### “Selectivity-Determining Region”: Residues +1, +3, and +5 Insert in Hydrophobic Pockets

**Figure 6:**
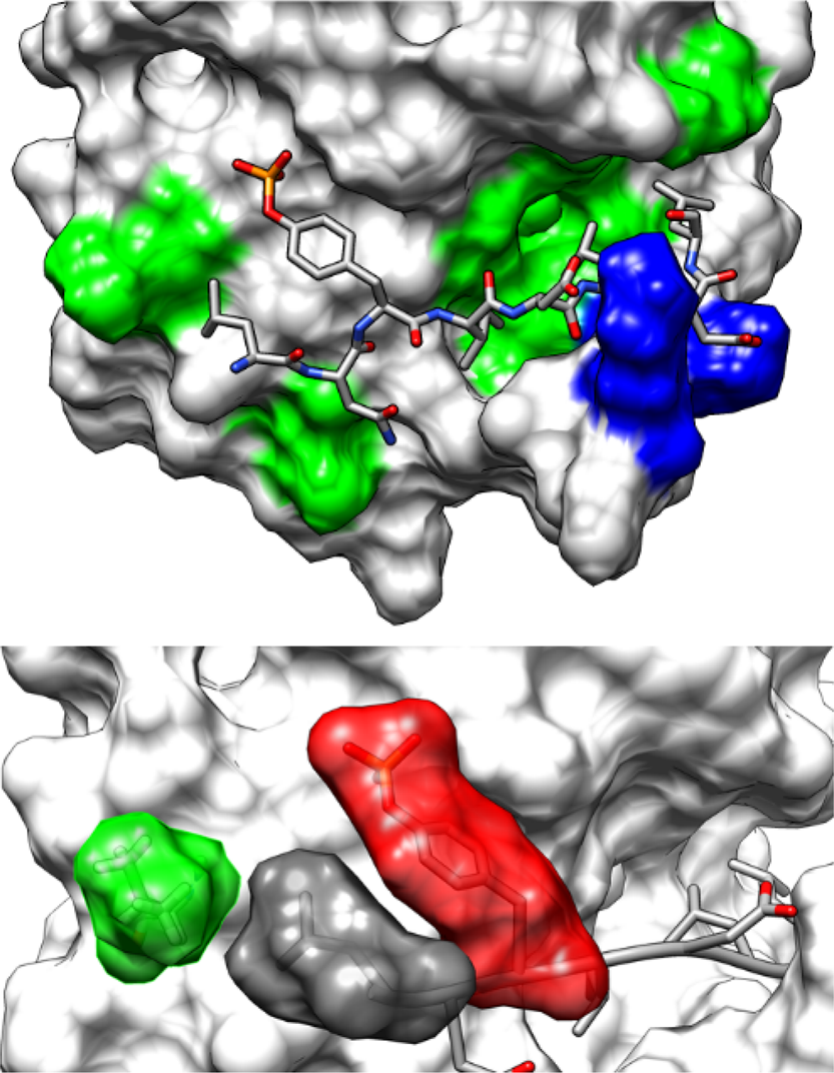
Most representative conformation in the IRS1-1172_8 MD simulation, illustrating the main specificity determining side-chain interactions. Top: hydrophobic regions of the domain surface are shown in green, while cationic K89 and K91 are reported in blue. Bottom: interactions of the L – 2 residue (gray surface), which inserts between the pY ring (red) and V14 (green).

Selectivity of SH2 domains is commonly considered to be determined mainly by residues C-terminal to the pY. Based on the interactions in this selectivity-determining region, the domains have been classified in three classes.^2,9,73,74^ The N-SH2 domain of SHP2 belongs to type II, called “open groove”, or “PLC-γ1-like”, in which residues C-terminal to the pY bind in a long hydrophobic groove, delimited by EF and BG loops. This is illustrated in Figure 6, which shows the most representative conformation of the IRS1-1172_8 MD simulation. With the pY inserted in its binding pocket, the extended conformation of the peptide backbone forces residues +1, +3, and +5 to point toward the protein core and to insert into the hydrophobic ridge. Residues +2 and +4 point toward the solvent but can interact with the loops BG and EF, which delimit the groove. In the N-terminal region of the peptide, residue −1 is solvent-exposed, while residue −2 points toward the protein surface in the region of helix αA.

Residues +1, +3, and +5 of the peptide (I, L, and L, respectively) form several interactions with hydrophobic amino acids that line the groove, remaining in contact with them for the whole length of the MD trajectory. In particular, residue +1 interacts with I54 (βD5), I96 (BG12), and methyl groups in the side chains of T52 (βD3) and E90 (BG6); residue +3 makes stable interactions with I54 (βD5), L65 (βE4), L88 (BG4), and I96 (BG12), and residue +5 interacts with L65 (βE4), Y81 (αB9), and L88 (BG4). Interestingly, the +1 pocket is the only one where polar residues are present in addition to hydrophobic amino acids. This might explain why peptide library studies and the sequences of known binding partners indicate that T can be present at position +1 of the peptide. As shown in Table 6, in the crystallographic structures 1AYA and 5DF6 (where a T is present in +1), no direct H-bond is formed between this residue and the protein domain. By contrast, our simulations show that a H-bond can indeed be formed either with T52 (βD3) or E90 (BG6). In one case (1AYA and PDGFR-1009), the peptide present in the crystal and in the simulation is the same one. However, the protein and peptide mobility, normally present in solution, can allow the formation of a H-bond that was not observed in the crystallographic structures.

**Table 6:** Hydrogen Bonds and Salt Bridges between Peptide Side Chains and the N-SH2 Domain^a^

		H-bonds					salt bridges			
method	ID	–1	+1	+2	+4	+6	–1 K35 (BC1)	+2 K91 (BG7)	+4 K89 (BG5)	+6 K91 (BG7)
PDB (Å)	4QSY	-	n.a.	-	-	**D-Oδ G68^N^ (EF3): 3.0**	n.a.	D:7.6	D:9.4	D:15
	1AYB	-	n.a.	-	-	n.a.	n.a.	n.a.	E:7.3	n.a.
	1AYA	n.a.	-	n.a.	-	n.a.	n.a.	n.a.	n.a.	n.a.
	5X94	n.a.	n.a.	-	-	-	n.a.	n.a.	n.a.	n.a.
	3TL0	-	n.a.	-	n.a.	n.a.	n.a.	n.a.	n.a.	n.a.
	5DF6	-	-	-	-	n.a.	n.a.	E:4.2	D:4.7	n.a.
	5X7B	n.a.	n.a.	-	-	n.a.	n.a.	n.a.	n.a.	n.a.
MD (%)	GAB1_10	*	n.a.	*	*	*	**E:58**	**D:62**	D:8	D:23
	GAB1_13	E-Oε H53^Nε^ (βD4): 42	n.a.	*	*	*	E:23	**D:81**	D:16	D:15
	IRS1-1172_8	N-Nδ V51^O^ (βD2): 24	n.a.	*	*	n.a.	n.a.	**D:83**	D:16	n.a.
	IRS1-1172_9	N-Nδ V51^O^ (βD2): 44	n.a.	*	*	n.a.	n.a.	**D:65**	D:22	n.a.
	IRS1-1172_11	-	n.a.	*	*	n.a.	n.a.	**D:71**	D:27	n.a.
	IRS1-1172_12	N-Nδ V51^O^ (βD2): 19	n.a.	*	*	n.a.	n.a.	**D:83**	D:14	n.a.
	IRS1-895	-	n.a.	-	E-Oε N92^N^ (BG8): 43	n.a.	-	n.a.	E:30 E:18 (K91)	n.a.
	IMHOF9	-	n.a.	Q-Oε K91^Nζ^ (BG7): 21	n.a	n.a.	n.a.	n.a.	n.a.	n.a.
	SWEENEY12	n.a.	n.a.	Q-Oδ K91^Nζ^ (BG7): 29	n.a.	n.a.	n.a.	n.a.	n.a.	n.a.
	IRS1-546	*	T-Oγ T52^Oγ^ (βD3): 24	*	n.a.	n.a.	E:31	**E:57**	n.a.	n.a.
	PDGFR-1009	n.a.	T-Oε E90^Oγ^ (BG6): 49	n.a.	Q-Nε E90^O^ (BG6): 22	**N-Nδ Q87^O^ (BG3): 62**	n.a.	n.a.	n.a.	n.a.
	IMHOF5	-	n.a.	Q-Oε K91^Nζ^ (BG7): 27	**W-Nε Q87^O^ (BG3): 91**	-	n.a.	n.a.	n.a.	n.a.

a The same protein residue numbering and definitions for stable interactions (reported in bold) of Table 5 were applied here. Distances (Å) and % persistence are reported for X-ray structures and MD simulations, respectively. Peptide residues are numbered with respect to the pY. n.a. indicates that the peptide residue is missing, or that the specific amino acid cannot form H-bonds/salt bridges, or that the protein residue (Lys 35, 89, or 91) was not resolved in the X-ray electron density. Dashes indicate that the H-bond is not formed in X-ray structures and that it is present for <5% in MD simulations; asterisks indicate that the H-bond is not reported because the same interaction was considered as an ion pair.

**Figure 7:**
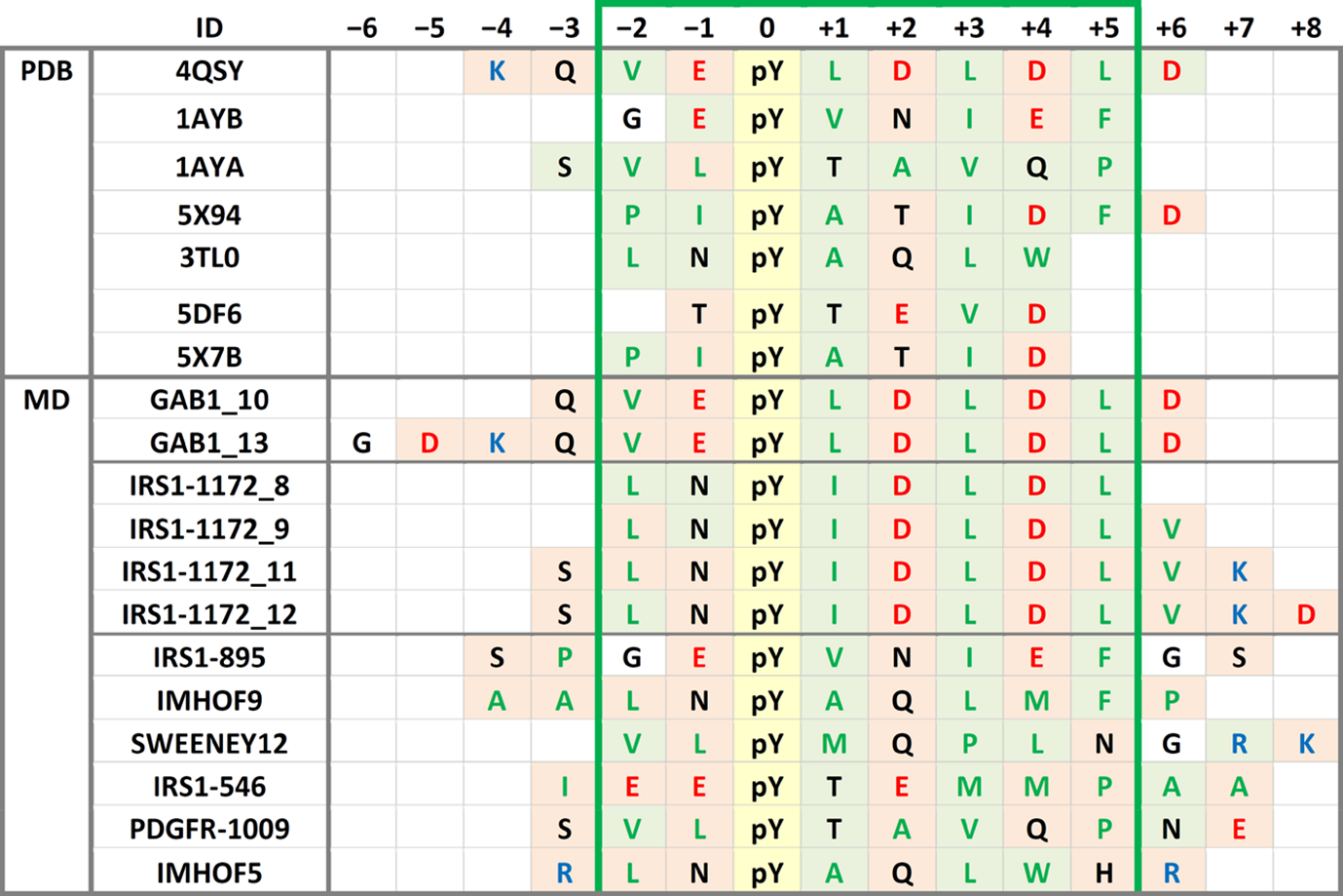
Solvent exposure of phosphopeptide residues; except for pY, each residue is colored in green when its solvent accessibility is lower than 50% and in red when it is higher than 50%. For MD simulations, an average value is reported. Hydrophobic, anionic, and cationic residues are colored in green, red, and blue, respectively.

To quantify the stability of the hydrophobic interactions between each peptide residue and the N-SH2 domain during all simulations, Figure 7 reports the solvent accessible surface (SAS) for each side chain. For comparison, the same parameter was calculated in the available crystallographic structures. Quantitative values are reported in Table S2. For all the simulated sequences, residues +1 and +3 remained stably embedded in the domain groove. Residue +5 was also buried in all cases where a hydrophobic side chain was present at that position (GAB1, IRS1-1172, IRS1-895, and IMHOF9 simulations, where residue +5 is L or F), with the single exception of the IRS1-1172_11 trajectory.

Overall, the hydrophobic interactions involving residues +1, +3, and +5 of the peptide, which characterize type II SH2 domains, remained stable in most of our simulations, corroborating their importance in determining the affinity and selectivity of the N-SH2 domain of SHP2.

### Characteristic Features of the SHP2 N-SH2 Domain: Interactions of Residues −2, −1, +2, and +4

The N-SH2 domain of SHP2, while part of class II, presents peculiar features, which could affect its binding selectivity. As discussed in the section focusing on the pY interactions, more than 80% of SH2 domains have a conserved arginine at position αA2. By contrast, the SH2 domains of SHP2, SHP1, and MATK have a glycine at that position.^3^ In the N-SH2 domain, the lack of side chain at position 13 (G αA2) favors the accessibility of an exposed V14 at position αA3. This peculiarity has been previously described^9,56,73^ and explains why the N-SH2 of SHP2 is one of the few SH2 domains in which residues N-terminal to the pY contribute to the binding specificity. Indeed, in several simulations, we observed that hydrophobic residues in −2 inserted between the pY ring and V14, interacting hydrophobically with both (Figure 6).

A second peculiarity, which has received limited attention in the literature, is that the N-SH2 domain has two K residues one amino acid apart in loop BG (K89 and K91, BG5 and BG7). The alignment of the human SH2 domains^3^ shows that positive residues in the BG loop are rather frequent and that 33 of the total 120 domains have a cationic amino acid in the position corresponding to K91. However, we noticed that a (K/R-X-K/R) pattern in the positions corresponding to K89 and K91, which face toward the peptide binding groove, is shared only by the SHP2 N-SH2 domain and by the C-terminal SH2 domains of PLC-γ1 and 2. In principle, these side chains could form electrostatic interactions with acidic residues present in +2 and +4 of the peptide, which are shown to be favorable at those positions by peptide array studies and by the sequences of high-affinity natural partners (Tables 1 and 2). In the available crystallographic structures, these interactions would be possible in 4QSY, 1AYB, and 5X94, where a D/E residue is present at positions +2, +4, or both. However, rather surprisingly, a bona fide ion pair is not formed in any of these structures (Table 6).

**Figure 8:**
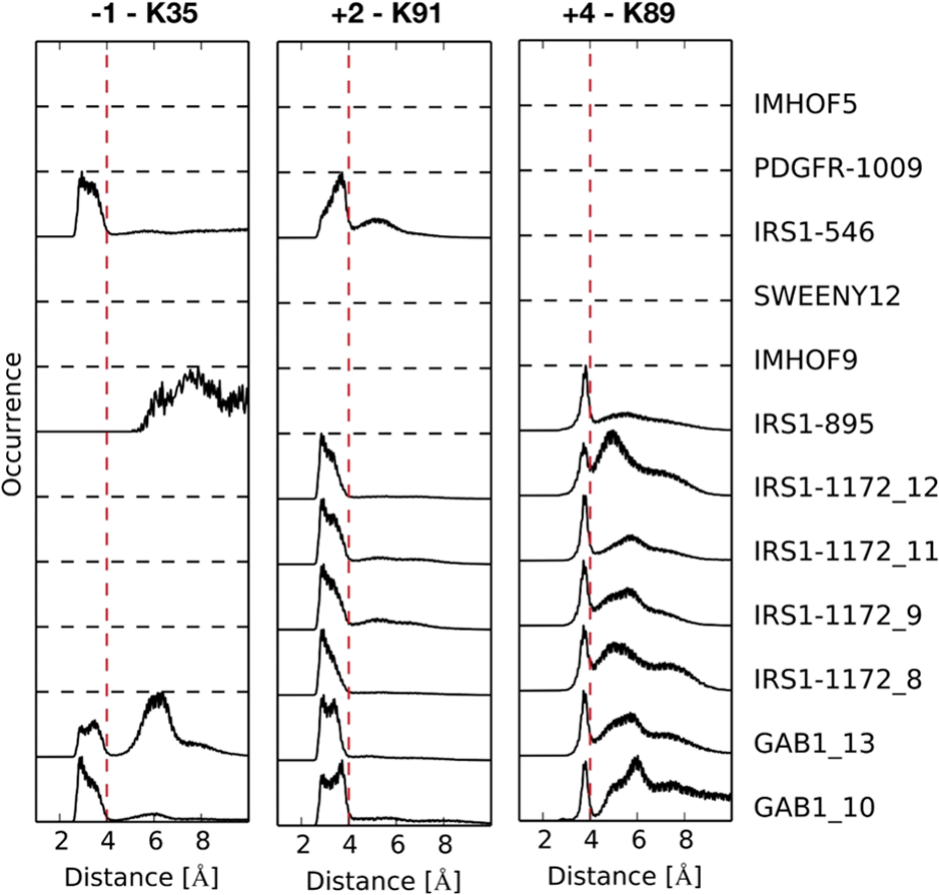
Most common intermolecular ion-pair interactions between the phosphopeptide side chains and the N-SH2 domain. Distribution of charged group distances populated in each MD trajectory. Distances of less than 4 Å (vertical red dashed lines) are indicative of a stable salt bridge. Dashed horizontal lines indicate that the corresponding phosphopeptide sequences lack an anionic residue at these positions and therefore cannot form the ion pair.

Among the simulated sequences, a D/E residue is present at position +2 or +4 (or both) in eight of the twelve simulations. Different from the X-ray conformations, our simulations show that a stable salt bridge forms between the +2 residue and K91 in all cases where this is possible. An ion pair between residues +4 and K89 forms, too, although only for a fraction of the simulation time (Table 6 and Figure 8). Interestingly, even polar, uncharged residues at positions +2 and +4 can interact with K89 and K91 by forming H-bonds (which, again, were not observed in the crystallographic structures). Therefore, the simulations indicate that electrostatic or H-bonding interactions between the BG loop and residues +2 and +4 can contribute significantly to the binding affinity and selectivity.

A third characteristic feature of the N-SH2 domain of SHP2 is the K residue at position BC1, which is present only in the C-terminal domain of ZAP70, while an R is present at that position in the N-SH2 domain of SHP1. As discussed above, in the crystallographic structures, K35 points toward the solvent. However, in the simulations, when E was present at position −1 (with the single exception of IRS-895), it interacted electrostatically with K35 (BC1). Interestingly, the trajectories in which this happened (GAB1 and IRS1-546) were the same in which the K35-pY ion pair was observed, as discussed above (Table 5). Probably, the negative residue in −1 favors a conformational transition, which brings the K35 side chain from being solvent-exposed to pointing toward the domain core, and in interaction with the pY, where it partially replaces K55 (Figure 5). A high mobility of K35 is supported by the observation that its side chain is not resolved in the electron density of several crystallographic structures (Table 5). In addition, during the simulations, polar residues in −1 could also form marginally stable H-bonds, with amino acids of the βD strand.

Finally, our simulations showed that some interactions are also possible for negatively charged or polar residues in +6. An aspartate in that position can interact electrostatically with K91 (BG7) (although without forming a stable ion pair due to the flexibility of the C-terminal end of the peptide). By contrast, in the crystallographic structure 4QSV, D +6 and K91 are very distant. In addition, the side chain of residue +6 can also form a H-bond with the EF or BG loops.

### The N-SH2 Domain Populates Different Conformations

**Figure 9:**
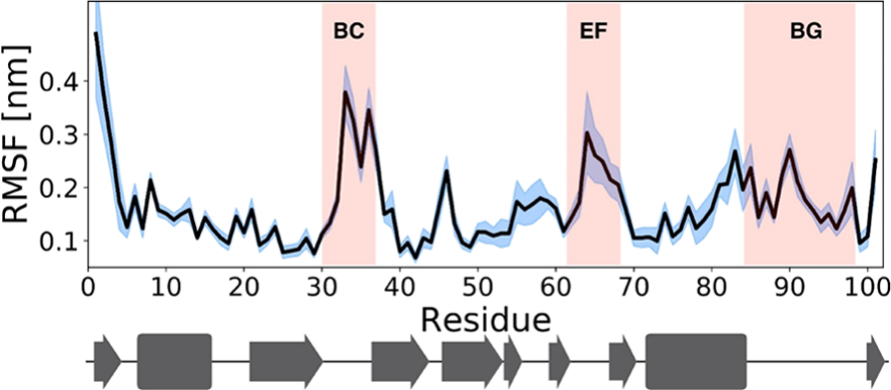
N-SH2 domain conformational variability in the MD simulations. Root-mean-square fluctuations (RMSF) of the N-SH2 domain backbone in the cumulative trajectory including all 12 simulations. The domain secondary structure is reported at the bottom for reference. The most mobile loops are highlighted in red in the figure. The blue-shaded area represents the standard deviation of the RMSF profile calculated between the twelve 1 μs trajectories.

The data reported above on interactions in the pY binding pocket in the MD simulations indirectly suggested a significant conformational variability of this region. This is clearly shown by an overall analysis of the domain mobility in the 12 trajectories. As shown in Figure 9, the most mobile regions were the BC loop, which forms the pY pocket, and the EF and BG loops, which control access to the hydrophobic specificity region.

**Figure 10:**
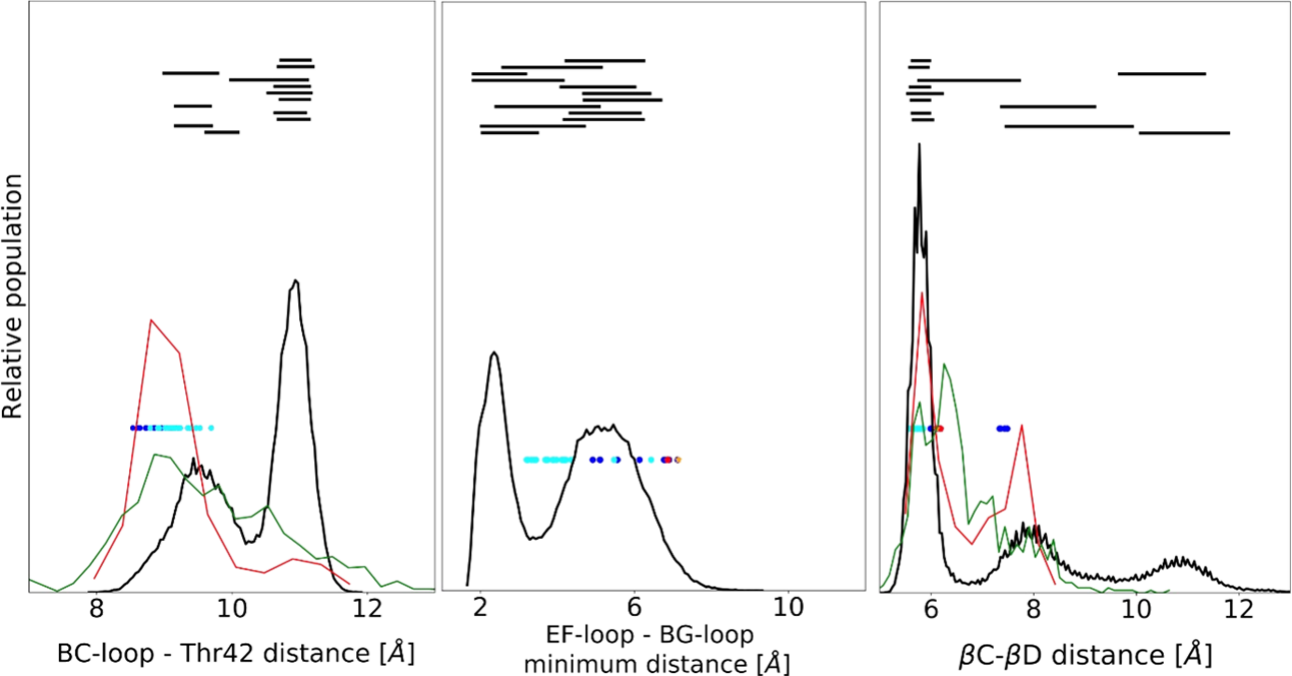
Structural parameters in simulated and experimental structures. Left: conformation of the pY pocket, as measured from the average distance between residues in the pY-loop (BC, residues 34–38) and T42 (βC3) in N-SH2 or structurally equivalent residues in other SH2 domains (see the Supporting Information). Center: conformation of the loops controlling access to the selectivity-determining region, as measured from the minimum distance between the EF loop (residues 66–69) and BG loop (residues 84–96). Right: conformation of the central β sheet as measured from the interstrand distance between the C atom of D40 (βC1) and N atom of Q57 (βD’1) or structurally equivalent residues in other SH2 domains. Data from the overall MD simulation of 12 N-SH2:peptide complexes are shown in black, along with analogous data from X-ray (red) and NMR (green) structures of SH2 domains. Values for experimental structures of isolated N-SH2 domains are shown as blue (when phosphopeptide-bound) or red dots (with no bound peptide). Values for structures of the domain in the whole SHP2 protein are reported as cyan (autoinhibited conformation) or orange dots (active conformation). Average ± standard deviation values of distances spanned by the individual simulations are indicated by black horizontal bars, reported in the order of Table 3, with GAB1_10 at the bottom and IMHOF5 at the top.

Figure 10 analyzes the conformation of these flexible regions. For the BC loop, it reports its average distance from T42, which is located in the pY pocket, on the βC strand (βC3) (Figure 10, left panel). While this loop is closed in all X-ray structures of the N-SH2 domain of SHP2, in our simulations, we find that it can change its structure significantly, populating also a more open conformation. Since this region is highly conserved in SH2 domains, we compared the MD conformations to those observed in experimental structures (both crystallographic and NMR, obtained in solution) of other SH2 domains. An open conformation of the BC loop is observed in only a few of the crystallographic structures but is significantly populated in solution according to NMR data. Therefore, our simulations might have observed a conformation of the pY loop that had not been previously reported for the N-SH2 domain of SHP2, possibly because it is disfavored by the crystal environment and by intermolecular crystallographic contacts.

The EF and BG loops, which regulate the accessibility of the specificity region, are distant in all structures of phosphopeptide/N-SH2 complexes and more closed in the structure of the autoinhibited state of SHP2. Indeed, based on structural data, this transition has been hypothesized to be part of the allosteric switch controlling SHP2 activity and binding affinity.^15,75,76^ In our simulations, we find that the loops can attain a significantly closed conformation even when a phosphopeptide is present in the binding cleft. In some trajectories (GAB1_10, GAB1_13, SWEENEY12, and IRS1-546), they stably embraced the peptide, clasping it tightly and getting in contact. The high sequence variability of the BG loop does not allow a quantitative comparison with the structures of other SH2 domains in this case. However, while such closed conformations have never been observed in X-ray structures of SHP2, for other SH2 domains, the EF and BG loops have been described as a ″set of jaws″ that clamp down on the peptide.^12,72^

**Figure 11:**
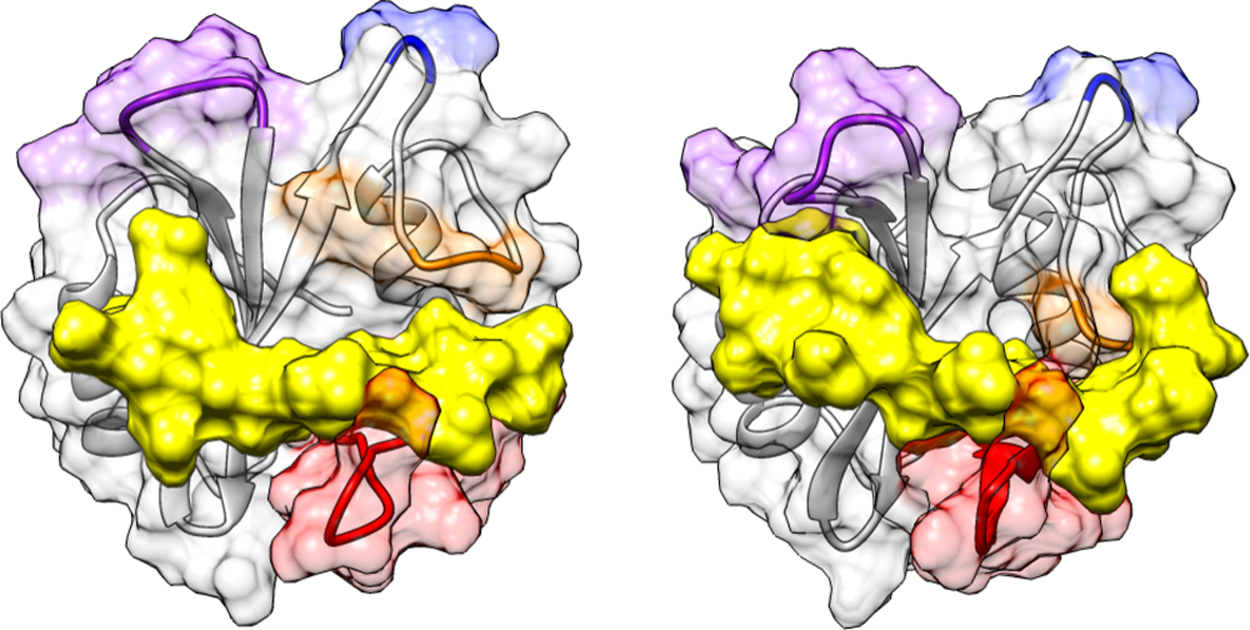
Conformational variability of the peptide-bound N-SH2 domain. Most representative structures of simulations IRS1-1172_9 and IRS1-1172_11, showing the conformational transitions of BC (purple), EF (orange), and BG (red) loops and of the central β sheet connecting strands C and D. The DE loop is highlighted in blue. The peptide surface is shown in yellow.

Another element of structural flexibility that we observed in our simulations is a variable length for the central β sheet. As shown in Figure 10, values going from ∼5 to ∼12 Å are populated for the distance between the N-terminal residue of the C strand (D40 and βC1) and the opposite residue in strand D (Q57 and βD’1). A similar variability (although in a smaller range) is present in the X-ray structures of the N-SH2 domain and also in the experimental structures of other SH2 domains. However, to the best of our knowledge, this important feature of conformational flexibility has not been previously discussed. These different conformational features are illustrated in Figure 11, which reports the most representative structures of two simulations.

As shown in Figure 10, each individual simulation populated only one region of the overall conformational space. This finding could be due to an effect of the peptide sequence on the conformational properties of the domain, but it could also be caused by insufficient sampling of the conformational space in the single simulations. Further studies will be required to clarify these aspects.

## Conclusions

This work analyzed the structural determinants of the binding affinity and selectivity of the N-SH2 domain of SHP2. Some of the features responsible for the sequence preferences of this domain were already visible in the previously published crystallographic structures. The simulations confirmed that, even in solution and notwithstanding the significant motions of the domain and of the bound peptide, these interactions are conserved. In particular, residues −2 to +5 are stably interacting with the domain, and this region of the peptide adopts an extended conformation (particularly from 0 to +3). The pY is stabilized in its pocket by multiple electrostatic and H-bonding interactions, while hydrophobic residues are needed at positions +1, +3, and +5, where they interact with apolar side chains of the domain binding groove.

These properties are common to type II SH2 domains. However, the simulations confirmed some peculiarities of the N-SH2 domain of SHP2, which differentiate it from other SH2 domains and might contribute to its selectivity. Specifically, in place of the commonly conserved R αA2, the N-SH2 domain of SHP2 has G13. As a consequence, a hydrophobic peptide residue at position −2 can insert in the space left free by the missing side chain and interact with the accessible side chain of V14 αA3, as well as with the phenol ring of pY, stabilizing its orientation and the overall complex. Indeed, selectivity for residues N-terminal to the pY is peculiar to the N-SH2 domain. Another characteristic property of the N-SH2 domain of SHP2 is the nonconserved T42 in βC3, which forms a stable H-bond with the pY phosphate.

More importantly, the simulations highlighted some features that were not visible in the crystal structures, thus providing novel insights into the binding preferences of the N-SH2 domain. A peculiarity of this domain is the K-X-K motif in the region of the BG loop facing toward the peptide binding groove. Anionic residues at positions +2 and +4 strongly interact with the two K side chains. Even polar amino acids at these positions in the peptide sequence can interact with them through H-bonds. These observations are supported by the frequent presence of acidic residues at these positions in the sequences of natural binding partners, while a similar sequence selectivity did not emerge clearly from peptide library studies.

Another feature characterizing the N-SH2 domain is that, in some cases, interactions extended up to residue +6 through H-bond or ion-pair formation with the EF or BG loops. This previously unexplored possibility warrants further investigation.

Polar amino acids at +1 can form H-bonds with residues in the corresponding domain pocket. This finding explains why a T residue was shown to be permitted at that position by library studies (in addition to hydrophobic amino acids).

Surprisingly, the conserved K βD6 does not form an ion pair with the pY phosphate in crystallographic structures. MD simulations indicated that, in solution, a slight rearrangement of the pY binding pocket might allow salt-bridge formation.

Another cationic residue is present in the pY pocket (K35, BC1), but in the crystallographic structures, it points toward the solvent, without interacting with the pY. Our simulations showed that the presence of an acidic residue at position −1 of the phosphopeptide can favor a conformational transition that brings K35 toward the domain. In this new orientation, it interacts both with pY and with the residue in −1.

Finally, we observed in our simulations a significant conformational flexibility of the domain. These conformational transitions were associated with the BC loop (which forms the pY pocket), with the DE and BG loops controlling access to the peptide binding groove and with the central βC and βB strands, and were broader than those previously hypothesized based on the different crystallographic structures of the domain. Investigation of the possible role of these motions in the function of SHP2 will require a more extensive exploration of the conformational properties of the N-SH2 domain.

## Supporting Information Available

PDB files for the most representative structure of each of the twelve simulations (ZIP)
Methods used for the analysis of experimental SH2 domain structures (Figure 10), Table S1 (preparation of the initial structures for the simulations), Table S2 (solvent accessibility values for the phosphopeptide side chains) (PDF)

## Abbreviations

SHP2: SH2-domain-containing tyrosine phosphate
SH2: Src homology 2 domain
pY: phosphotyrosine
MD: molecular dynamics
JMML: juvenile myelomonocytic leukemia
Erk: extracellular signal-regulated kinases
MAPK: mitogen-activated protein kinase
RTK: receptor tyrosine kinase
IRS-1: insulin receptor substrate 1
PDGFR: platelet-derived growth factor receptor
CagA: cytotoxin-associated gene A
Gab1: GRB2-associated binding protein
PLC-γ1: phospholipase C gamma 1
ZAP70: zeta-chain-associated protein kinase 70
MOE: molecular operative environment
PME: particle-mesh Ewald
RMSF: root-mean-square fluctuations

## References

[ref1] Sadowski I.; Stone J. C.; Pawson T. A noncatalytic domain conserved among cytoplasmic protein-tyrosine kinases modifies the kinase function and transforming activity of Fujinami sarcoma virus P130gag-fps. Mol. Cell. Biol. 1986, 6, 4396–4408. 10.1128/MCB.6.12.4396. 3025655 PMC367222

[ref2] Yaffe M. B. Phosphotyrosine-binding domains in signal transduction. Nat. Rev. Mol. Cell Biol. 2002, 3, 177–186. 10.1038/nrm759. 11994738

[ref3] Liu B. A.; Jablonowski K.; Raina M.; Arcé M.; Pawson T.; Nash P. D. The human and mouse complement of SH2 domain proteins—establishing the boundaries of phosphotyrosine signaling. Mol. Cell 2006, 22, 851–868. 10.1016/j.molcel.2006.06.001. 16793553

[ref4] Liu B. A.; Engelmann B. W.; Nash P. D. The language of SH2 domain interactions defines phosphotyrosine-mediated signal transduction. FEBS Lett. 2012, 586, 2597–2605. 10.1016/j.febslet.2012.04.054. 22569091

[ref5] Mayer B. J. What Have We Learned from SH2 Domains? In SH2 Domains; Liu B. A., Machida K., Eds.; Humana Press: New York, 2017; pp 37–43.

[ref6] Gopalasingam P.; Quill L.; Jeeves M.; Overduin M. SH2 domain structures and interactions. In SH Domains; Kurochkina N., Ed.: Springer: Cham, 2015; pp. 159–185, 10.1007/978-3-319-20098-9_8.

[ref7] Pawson T.; Scott J. D. Signaling through scaffold, anchoring, and adaptor proteins. Science 1997, 278, 2075–2080. 10.1126/science.278.5346.2075. 9405336

[ref8] Kaneko T.; Joshi R.; Feller S. M.; Li S. S. Phosphotyrosine recognition domains: the typical, the atypical and the versatile. Cell Commun. Signaling 2012, 10, 32 10.1186/1478-811X-10-32. PMC3507883 23134684

[ref9] Bradshaw J. M.; Waksman G. Molecular recognition by SH2 domains. Adv. Protein Chem. 2002, 61, 161–210. 10.1016/S0065-3233(02)61005-8. 12461824

[ref10] Jadwin J. A.; Curran T. G.; Lafontaine A. T.; White F. M.; Mayer B. J. Src homology 2 domains enhance tyrosine phosphorylation in vivo by protecting binding sites in their target proteins from dephosphorylation. J. Biol. Chem. 2018, 293, 623–637. 10.1074/jbc.M117.794412. 29162725 PMC5767867

[ref11] Liu B. A.; Machida K. Introduction: History of SH2 Domains and Their Applications. In SH2 Domains; Liu B. A., Machida K., Eds.; Humana Press: New York, 2017; pp 3–35. 10.1007/978-1-4939-6762-9_1 28092024

[ref12] Eck M. J.; Shoelson S. E.; Harrison S. C. Recognition of a high-affinity phosphotyrosyl peptide by the Src homology-2 domain of p56 lck. Nature 1993, 362, 87–91. 10.1038/362087a0. 7680435

[ref13] Kuriyan J.; Cowburn D. Modular peptide recognition domains in eukaryotic signaling. Annu. Rev. Biophys. Biomol. Struct. 1997, 26, 259–288. 10.1146/annurev.biophys.26.1.259. 9241420

[ref14] Mayer B. J.; Gupta R. Functions of SH2 and SH3 domains. In Protein Modules in Signal Transduction; Pawson A. J., Ed.; Springer: Berlin, 1998; pp 1–22. 10.1007/978-3-642-80481-6_1 9401200

[ref15] Hof P.; Pluskey S.; Dhe-Paganon S.; Eck M. J.; Shoelson S. E. Crystal structure of the tyrosine phosphatase SHP-2. Cell 1998, 92, 441–450. 10.1016/S0092-8674(00)80938-1. 9491886

[ref16] Tartaglia M.; Niemeyer C. M.; Fragale A.; Song X.; Buechner J.; Jung A.; Hählen K.; Hasle H.; Licht J. D.; Gelb B. D. Somatic mutations in PTPN11 in juvenile myelomonocytic leukemia, myelodysplastic syndromes and acute myeloid leukemia. Nat. Genet. 2003, 34, 148–150. 10.1038/ng1156. 12717436

[ref17] Tartaglia M.; Martinelli S.; Cazzaniga G.; Cordeddu V.; Iavarone I.; Spinelli M.; Palmi C.; Carta C.; Pession A.; Aricò M.; Masera G.; Basso G.; Sorcini M.; Gelb B. D.; Biondi A. Genetic evidence for lineage-related and differentiation stage–related contribution of somatic PTPN11 mutations to leukemogenesis in childhood acute leukemia. Blood 2004, 104, 307–313. 10.1182/blood-2003-11-3876. 14982869

[ref18] Tartaglia M.; Niemeyer C. M.; Shannon K. M.; Loh M. L. SHP-2 and myeloid malignancies. Curr. Opin. Hematol. 2004, 11, 44–50. 10.1097/00062752-200401000-00007. 14676626

[ref19] Grossmann K. S.; Rosário M.; Birchmeier C.; Birchmeier W. The tyrosine phosphatase Shp2 in development and cancer. In Advances in cancer research; Vande Woude G. F., Klein G., Eds.; Academic Press: San Diego, 2010; Vol. 106, pp. 53–89. 10.1016/S0065-230X(10)06002-1 20399956

[ref20] Chen Y. N. P.; LaMarche M. J.; Chan H. M.; Fekkes P.; Garcia-Fortanet J.; Acker M. G.; Antonakos B.; Chen C. H.-T.; Chen Z.; Cooke V. G.; Dobson J. R.; Deng Z.; Fei F.; Firestone B.; Fodor M.; Fridrich C.; Gao H.; Grunenfelder D.; Hao H. X.; Jacob J.; Ho S.; Hsiao K.; Kang Z. B.; Karki R.; Kato M.; Larrow J.; La Bonte L. R.; Lenoir F.; Liu G.; Liu S.; Majumdar D.; Meyer M. J.; Palermo M.; Perez L.; Pu M.; Price E.; Quinn C.; Shakya S.; Shultz M. D.; Slisz J.; Venkatesan K.; Wang P.; Warmuth M.; Williams S.; Yang G.; Yuan J.; Zhang J. H.; Zhu P.; Ramsey T.; Keen N. J.; Sellers W. R.; Stams T.; Fortin P. D. Allosteric inhibition of SHP2 phosphatase inhibits cancers driven by receptor tyrosine kinases. Nature 2016, 535, 148–152. 10.1038/nature18621. 27362227

[ref21] Ahmed T. A.; Adamopoulos C.; Karoulia Z.; Wu X.; Sachidanandam R.; Aaronson S. A.; Poulikakos P. I. SHP2 Drives Adaptive Resistance to ERK Signaling Inhibition in Molecularly Defined Subsets of ERK-Dependent Tumors. Cell Rep. 2019, 26, 65–78.e5. 10.1016/j.celrep.2018.12.013. 30605687 PMC6396678

[ref22] Okazaki T.; Chikuma S.; Iwai Y.; Fagarasan S.; Honjo T. A rheostat for immune responses: the unique properties of PD-1 and their advantages for clinical application. Nat. Immunol. 2013, 14, 1212 10.1038/ni.2762. 24240160

[ref23] Hayashi T.; Senda M.; Suzuki N.; Nishikawa H.; Ben C.; Tang C.; Nagase L.; Inoue K.; Senda T.; Hatakeyama M. Differential mechanisms for SHP2 binding and activation are exploited by geographically distinct Helicobacter pylori CagA oncoproteins. Cell Rep. 2017, 20, 2876–2890. 10.1016/j.celrep.2017.08.080. 28930683

[ref24] Tartaglia M.; Mehler E. L.; Goldberg R.; Zampino G.; Brunner H. G.; Kremer H.; van der Burgt I.; Crosby A. H.; Ion A.; Jeffery S.; Kalidas K.; Patton M. A.; Kucherlapati R. S.; Gelb B. D. Mutations in PTPN11, encoding the protein tyrosine phosphatase SHP-2, cause Noonan syndrome. Nat. Genet. 2001, 29, 465–468. 10.1038/ng772. 11704759

[ref25] Tartaglia M.; Gelb B. D. Disorders of dysregulated signal traffic through the RAS-MAPK pathway: phenotypic spectrum and molecular mechanisms. Ann. N. Y. Acad. Sci. 2010, 1214, 99 10.1111/j.1749-6632.2010.05790.x. 20958325 PMC3010252

[ref26] Butterworth S.; Overduin M.; Barr A. J. Targeting protein tyrosine phosphatase SHP2 for therapeutic intervention. Future Med. Chem. 2014, 6, 1423–1437. 10.4155/fmc.14.88. 25329198

[ref27] Ran H.; Tsutsumi R.; Araki T.; Neel B. G. Sticking it to cancer with molecular glue for SHP2. Cancer Cell 2016, 30, 194–196. 10.1016/j.ccell.2016.07.010. 27505669 PMC5558882

[ref28] Frankson R.; Yu Z. H.; Bai Y.; Li Q.; Zhang R. Y.; Zhang Z. Y. Therapeutic targeting of oncogenic tyrosine phosphatases. Cancer Res. 2017, 77, 5701–5705. 10.1158/0008-5472.CAN-17-1510. 28855209 PMC5827927

[ref29] Tartaglia M.; Martinelli S.; Stella L.; Bocchinfuso G.; Flex E.; Cordeddu V.; Zampino G.; van der Burgt I.; Palleschi A.; Petrucci T. C.; Sorcini M.; Schoch C.; Foá R.; Emanuel P. D.; Gelb B. D. Diversity and functional consequences of germline and somatic PTPN11 mutations in human disease. Am. J. Hum. Genet. 2006, 78, 279–290. 10.1086/499925. 16358218 PMC1380235

[ref30] Bocchinfuso G.; Stella L.; Martinelli S.; Flex E.; Carta C.; Pantaleoni F.; Pispisa B.; Venanzi M.; Tartaglia M.; Palleschi A. Structural and functional effects of disease-causing amino acid substitutions affecting residues Ala72 and Glu76 of the protein tyrosine phosphatase SHP-2. Proteins: Struct., Funct., Bioinf. 2007, 66, 963–974. 10.1002/prot.21050. 17177198

[ref31] Martinelli S.; Torreri P.; Tinti M.; Stella L.; Bocchinfuso G.; Flex E.; Grottesi A.; Ceccarini M.; Palleschi A.; Cesareni G.; Castagnoli L.; Petrucci T. C.; Gelb B. D.; Tartaglia M. Diverse driving forces underlie the invariant occurrence of the T42A, E139D, I282V and T468M SHP2 amino acid substitutions causing Noonan and LEOPARD syndromes. Hum. Mol. Genet. 2008, 17, 2018–2029. 10.1093/hmg/ddn099. 18372317 PMC2900904

[ref32] Martinelli S.; Nardozza A. P.; Delle Vigne S.; Sabetta G.; Torreri P.; Bocchinfuso G.; Flex E.; Venanzi S.; Palleschi A.; Gelb B. D.; Cesareni G.; Stella L.; Castagnoli L.; Tartaglia M. Counteracting effects operating on Src homology 2 domain-containing protein-tyrosine phosphatase 2 (SHP2) function drive selection of the recurrent Y62D and Y63C substitutions in Noonan syndrome. J. Biol. Chem. 2012, 287, 27066–27077. 10.1074/jbc.M112.350231. 22711529 PMC3411048

[ref33] Machida K.; Mayer B. J. The SH2 domain: versatile signaling module and pharmaceutical target. Biochim. Biophys. Acta, Proteins Proteomics 2005, 1747, 1–25. 10.1016/j.bbapap.2004.10.005. 15680235

[ref34] Songyang Z.; Shoelson S. E.; Chaudhuri M.; Gish G.; Pawson T.; Haser W. G.; King F.; Roberts T.; Ratnofsky S.; Lechleider R. J.; Neel B. G.; Birge R. B.; Fajardo J. E.; Chou M. M.; Hanafusa H.; Schaffhausen B.; Cantley L. C. SH2 domains recognize specific phosphopeptide sequences. Cell 1993, 72, 767–778. 10.1016/0092-8674(93)90404-e. 7680959

[ref35] Sweeney M. C.; Wavreille A. S.; Park J.; Butchar J. P.; Tridandapani S.; Pei D. Decoding protein-protein interactions through combinatorial chemistry: sequence specificity of SHP-1, SHP-2, and SHIP SH2 domains. Biochemistry 2005, 44, 14932–14947. 10.1021/bi051408h. 16274240

[ref36] Imhof D.; Wavreille A. S.; May A.; Zacharias M.; Tridandapani S.; Pei D. Sequence Specificity of SHP-1 and SHP-2 Src Homology 2 Domains: Critical Roles of Residues Beyond the pY+ 3 Position. J. Biol. Chem. 2006, 281, 20271–20282. 10.1074/jbc.M601047200. 16702225

[ref37] Yang X.; Dutta U.; Shaw L. M. SHP2 mediates the localized activation of Fyn downstream of the α6β4 integrin to promote carcinoma invasion. Mol. Cell. Biol. 2010, 30, 5306–5317. 10.1128/MCB.00326-10. 20855525 PMC2976378

[ref38] Kumamaru E.; Numakawa T.; Adachi N.; Kunugi H. Glucocorticoid suppresses BDNF-stimulated MAPK/ERK pathway via inhibiting interaction of Shp2 with TrkB. FEBS Lett. 2011, 585, 3224–3228. 10.1016/j.febslet.2011.09.010. 21946312

[ref39] Tsutsumi R.; Masoudi M.; Takahashi A.; Fujii Y.; Hayashi T.; Kikuchi I.; Satou Y.; Taira M.; Hatakeyama M. YAP and TAZ, Hippo signaling targets, act as a rheostat for nuclear SHP2 function. Dev. Cell 2013, 26, 658–665. 10.1016/j.devcel.2013.08.013. 24035415

[ref40] Gandji L. Y.; Proust R.; Larue L.; Gesbert F. The tyrosine phosphatase SHP2 associates with CUB domain-containing protein-1 (CDCP1), regulating its expression at the cell surface in a phosphorylation-dependent manner. PLoS One 2015, 10, e0123472 10.1371/journal.pone.0123472. 25876044 PMC4395315

[ref41] Breitkopf S. B.; Yang X.; Begley M. J.; Kulkarni M.; Chiu Y. H.; Turke A. B.; Lauriol J.; Yuan M.; Qi J.; Engelman J. A.; Hong P.; Kontaridis M. I.; Cantley L. C.; Perrimon N.; Asara J. M. A cross-species study of PI3K protein-protein interactions reveals the direct interaction of P85 and SHP2. Sci. Rep. 2016, 6, 1–14. 10.1038/srep20471. 26839216 PMC4738311

[ref42] Müller P. J.; Rigbolt K. T. G.; Paterok D.; Piehler J.; Vanselow J.; Lasonder E.; Andersen J. S.; Schaper F.; Sobota R. M. Protein tyrosine phosphatase SHP2/PTPN11 mistargeting as a consequence of SH2-domain point mutations associated with Noonan Syndrome and leukemia. J. Proteomics 2013, 84, 132–147. 10.1016/j.jprot.2013.04.005. 23584145

[ref43] Vemulapalli V.; Chylek L.; Erickson A.; LaRochelle J.; Subramanian K.; Mohseni M.; LaMarche M.; Acker M. G.; Sorger P. K.; Gygi S. P.; Blacklow S. C. Time resolved quantitative phosphoproteomics reveals distinct patterns of SHP2 dependence in EGFR signaling. bioRxiv 2019, 598664 10.1101/598664.

[ref44] Koncz G.; Tóth G. K.; Bökönyi G.; Kéri G.; Pecht I.; Medgyesi D.; Gergely J.; Sármay G. Co-clustering of Fcγ and B cell receptors induces dephosphorylation of the Grb2-associated binder 1 docking protein. Eur. J. Biochem. 2001, 268, 3898–3906. 10.1046/j.1432-1327.2001.02295.x. 11453982

[ref45] Sugimoto S.; Wandless T. J.; Shoelson S. E.; Neel B. G.; Walsh C. T. Activation of the SH2-containing protein tyrosine phosphatase, SH-PTP2, by phosphotyrosine-containing peptides derived from insulin receptor substrate-1. J. Biol. Chem. 1994, 269, 13614–13622. 7513703

[ref46] Bonetti D.; Troilo F.; Toto A.; Travaglini-Allocatelli C.; Brunori M.; Gianni S. Mechanism of Folding and Binding of the N-Terminal SH2 Domain from SHP2. J. Phys. Chem B 2018, 122, 11108–11114. 10.1021/acs.jpcb.8b05651. 30047735

[ref47] Case R. D.; Piccione E.; Wolf G.; Benett A. M.; Lechleider R. J.; Neel B. G.; Shoelson S. E. SH-PTP2/Syp SH2 domain binding specificity is defined by direct interactions with platelet-derived growth factor beta-receptor, epidermal growth factor receptor, and insulin receptor substrate-1-derived phosphopeptides. J. Biol. Chem. 1994, 269, 10467–10474. 8144631

[ref48] Huyer G.; Li Z. M.; Adam M.; Huckle W. R.; Ramachandran C. Direct determination of the sequence recognition requirements of the SH2 domains of SH-PTP2. Biochemistry 1995, 34, 1040–1049. 10.1021/bi00003a039. 7530043

[ref49] Takada T.; Matozaki T.; Takeda H.; Fukunaga K.; Noguchi T.; Fujioka Y.; Okazaki I.; Tsuda M.; Yamao T.; Ochi F.; Kasuga M. Roles of the complex formation of SHPS-1 with SHP-2 in insulin-stimulated mitogen-activated protein kinase activation. J. Biol. Chem. 1998, 273, 9234–9242. 10.1074/jbc.273.15.9234. 9535915

[ref50] Rönnstrand L.; Arvidsson A. K.; Kallin A.; Rorsman C.; Hellman U.; Engström U.; Wernstedt C.; Heldin C. H. SHP-2 binds to Tyr763 and Tyr1009 in the PDGF β-receptor and mediates PDGF-induced activation of the Ras/MAP kinase pathway and chemotaxis. Oncogene 1999, 18, 3696–3702. 10.1038/sj.onc.1202705. 10391677

[ref51] Ottinger E. A.; Botfield M. C.; Shoelson S. E. Tandem SH2 domains confer high specificity in tyrosine kinase signaling. J. Biol. Chem. 1998, 273, 729–735. 10.1074/jbc.273.2.729. 9422724

[ref52] Huang H.; Li L.; Wu C.; Schibli D.; Colwill K.; Ma S.; Li C.; Roy P.; Ho K.; Songyang Z.; Pawson T.; Gao Y.; Li S. S.-C. Defining the specificity space of the human SRC homology 2 domain. Mol. Cell Proteomics 2008, 7, 768–784. 10.1074/mcp.M700312-MCP200. 17956856

[ref53] Tinti M.; Kiemer L.; Costa S.; Miller M. L.; Sacco F.; Olsen J. V.; Carducci M.; Paoluzi S.; Langone F.; Workman C. T.; Blom N.; Machida K.; Thompson C. M.; Schutkowski M.; Brunak S.; Mann M.; Mayer B. J.; Castagnoli L.; Cesareni G. The SH2 domain interaction landscape. Cell Rep. 2013, 3, 1293–1305. 10.1016/j.celrep.2013.03.001. 23545499 PMC4110347

[ref54] De Souza D.; Fabri L. J.; Nash A.; Hilton D. J.; Nicola N. A.; Baca M. SH2 domains from suppressor of cytokine signaling-3 and protein tyrosine phosphatase SHP-2 have similar binding specificities. Biochemistry 2002, 41, 9229–9236. 10.1021/bi0259507. 12119038

[ref55] Zhang Y.; Zhang J.; Yuan C.; Hard R. L.; Park I. H.; Li C.; et al. Simultaneous binding of two peptidyl ligands by a Src homology 2 domain. Biochemistry 2011, 50, 7637–7646. 10.1021/bi200439v. 21800896 PMC3164524

[ref56] Lee C. H.; Kominos D.; Jacques S.; Margolis B.; Schlessinger J.; Shoelson S. E.; Kuriyan J. Crystal structures of peptide complexes of the amino-terminal SH2 domain of the Syp tyrosine phosphatase. Structure 1994, 2, 423–438. 10.1016/S0969-2126(00)00044-7. 7521735

[ref57] Case D. A.; Darden T. A.; Cheatham T.E. III; Simmerling C.L.; Wang J.; Duke R.E.; Luo R.; Walker R.C.; Zhang W.; Merz K.M.; Roberts B. P.; Hayik S.; Roitberg A.; Seabra G.; Swails J.; Götz A. W.; Kolossváry I.; Wong K. F.; Paesani F.; Vanicek J.; Wolf R. M.; Liu J.; Wu X.; Brozell S. R.; Steinbrecher T.; Gohlke H.; Cai Q.; Ye X.; Wang J.; Hsieh M. J.; Cui G.; Roe D. R.; Mathews D. H.; Seetin M. G.; Salomon-Ferrer R.; Sagui C.; Babin V.; Luchko T.; Gusarov S.; Kovalenko A.; Kollman P. A. AMBER 12; University of California: San Francisco, 2012.

[ref58] Liu Y.; Lau J.; Li W.; Tempel W.; Li L.; Dong A.; Narula A.; Qin S.; Min J. Structural basis for the regulatory role of the PPxY motifs in the thioredoxin-interacting protein TXNIP. Biochem. J. 2016, 473, 179–187. 10.1042/BJ20150830. 26527736

[ref59] Jorgensen W. L.; Chandrasekhar J.; Madura J. D.; Impey R. W.; Klein M. L. Comparison of simple potential functions for simulating liquid water. J. Chem. Phys. 1983, 79, 926–935. 10.1063/1.445869.

[ref60] Van Der Spoel D.; Lindahl E.; Hess B.; Groenhof G.; Mark A. E.; Berendsen H. J. C. GROMACS: fast, flexible, and free. J. Comput. Chem. 2005, 26, 1701–1718. 10.1002/jcc.20291. 16211538

[ref61] Hornak V.; Abel R.; Okur A.; Strockbine B.; Roitberg A.; Simmerling C. Comparison of multiple Amber force fields and development of improved protein backbone parameters. Proteins: Struct., Funct., Bioinf. 2006, 65, 712–725. 10.1002/prot.21123. PMC4805110 16981200

[ref62] Homeyer N.; Horn A. H. C.; Lanig H.; Sticht H. AMBER force-field parameters for phosphorylated amino acids in different protonation states: phosphoserine, phosphothreonine, phosphotyrosine, and phosphohistidine. J. Mol. Model. 2006, 12, 281–289. 10.1007/s00894-005-0028-4. 16240095

[ref63] Darden T.; York D.; Pedersen L. Particle mesh Ewald: An N·log(N) method for Ewald sums in large systems. J. Chem. Phys. 1993, 98, 10089–10092. 10.1063/1.464397.

[ref64] Miyamoto S.; Kollman P. A. Settle: An analytical version of the SHAKE and RATTLE algorithm for rigid water models. J. Comput. Chem 1992, 13, 952–962. 10.1002/jcc.540130805.

[ref65] Hess B.; Bekker H.; Berendsen H. J. C.; Fraaije J. G. E. M. LINCS: a linear constraint solver for molecular simulations. J. Comput. Chem. 1997, 18, 1463–1472. 10.1002/(SICI)1096-987X(199709)18:12<1463::AID-JCC4>3.0.CO;2-H.

[ref66] Berendsen H. J. C.; Postma J. P. M.; van Gunsteren W. F.; DiNola A.; Haak J. R. Molecular dynamics with coupling to an external bath. J. Chem. Phys. 1984, 81, 3684–3690. 10.1063/1.448118.

[ref67] Bussi G.; Donadio D.; Parrinello M. Canonical sampling through velocity rescaling. J. Chem. Phys. 2007, 126, 014101 10.1063/1.2408420. 17212484

[ref68] Mills J. E. J.; Dean P. M. Three-dimensional hydrogen-bond geometry and probability information from a crystal survey. J. Comput.-Aided Mol. Des. 1996, 10, 607–622. 10.1007/BF00134183. 9007693

[ref69] Hyberts S. G.; Goldberg M. S.; Havel T. F.; Wagner G. The solution structure of eglin c based on measurements of many NOEs and coupling constants and its comparison with X-ray structures. Protein Sci. 1992, 1, 736–751. 10.1002/pro.5560010606. 1304915 PMC2142248

[ref70] Cantor C. R.; Schimmel P. R. Biophysical Chemistry, Part I: The Conformation of Biological Molecules*;* W H Freeman & Co: San Francisco, 1980.

[ref71] Ramakrishnan C. Ramachandran and his map. Resonance 2001, 6, 48–56. 10.1007/BF02836967.

[ref72] Cohen G. B.; Ren R.; Baltimore D. Modular binding domains in signal transduction proteins. Cell 1995, 80, 237–248. 10.1016/0092-8674(95)90406-9. 7834743

[ref73] Waksman G.; Kuriyan J. Structure and specificity of the SH2 domain. Cell 2004, 116, S45–S51. 10.1016/S0092-8674(04)00043-1. 15055581

[ref74] Roque A. C. A.; Lowe C. R. Lessons from nature: On the molecular recognition elements of the phosphoprotein binding-domains. Biotechnol. Bioeng. 2005, 91, 546–555. 10.1002/bit.20561. 15959902

[ref75] Barford D.; Neel B. G. Revealing mechanisms for SH2 domain mediated regulation of the protein tyrosine phosphatase SHP-2. Structure 1998, 6, 249–254. 10.1016/S0969-2126(98)00027-6. 9551546

[ref76] Darian E.; Guvench O.; Yu B.; Qu C. K.; MacKerell A. D. Jr. Structural mechanism associated with domain opening in gain-of-function mutations in SHP2 phosphatase. Proteins: Struct., Funct., Bioinf. 2011, 79, 1573–1588. 10.1002/prot.22984. PMC3076527 21365683

