# Supplementary material for: Structural Determinants of Phosphopeptide Binding to the N-Terminal Src Homology 2 Domain of the SHP2 Phosphatase
Source: J Chem Inf Model. 2020 May 12;60(6):3157–71. doi: 10.1021/acs.jcim.0c00307 (PMC8007070; doi:10.1021/acs.jcim.0c00307)
Supplement: Supplementary file 2 — ci0c00307_si_002.pdf [file ci0c00307_si_002.pdf]

# SUPPORTING INFORMATION FOR

# Structural Determinants of Phosphopeptide Binding to the N-Terminal Src Homology 2 Domain of the SHP2 Phosphatase

*Massimiliano Anselmi,[1,‡,†] Paolo Calligari,[1,‡] Jochen S. Hub,[2] Marco Tartaglia,[3] Gianfranco Bocchinfuso,[1,*] Lorenzo Stella.[1,*]*

*1 Department of Chemical Sciences and Technologies, University of Rome Tor Vergata, 00133, Rome, Italy.*

*2 Theoretical Physics and Center for Biophysics, Saarland University, Campus E2 6, 66123 Saarbrücken, Germany.*

*3 Genetics and Rare Diseases Research Division, Ospedale Pediatrico Bambino Gesù, IRCCS, 00146 Rome, Italy.*

‡*contributed equally*

* *to whom correspondence should be addressed*

ANALYSIS OF AVAILABLE EXPERIMENTAL STRUCTURES OF SH2 DOMAINS

Two structural variables discussed for the MD simulations of the N-SH2 domain of SHP2 (Figure 9) were calculated also for the available experimental structures of SH2 domains. In the case of the N-SH2 domain, these variables were defined as:

- the "opening" of the central β-sheet, measured as the distance between $C_\alpha$ of residues Asp40 (strand β2) and Gln57 (strand β3);
- the "opening" of the pY-loop, measured as the average distance between $C_\alpha$ of Thr42 (strand β2) and $C_\alpha$ atoms of the five central residues belonging to the pY-loop (Ser34, Lys35, Ser36, Asn37, Pro38).

In the case of the experimental structures of SH2 domains, each structure was superimposed to 1AYD (X-ray structure of unbound SHP2 N-SH2), using the "matchmaker" function of UCSF Chimera [Pettersen, E. F.; Goddard, T. D.; Huang, C. C.; Couch, G. S.; Greenblatt, D. M.; Meng, E. C.; Ferrin, T. E. UCSF Chimera—a visualization system for exploratory research and analysis. *J. Comp. Chem.* **2004**, *25*, 1605-1612.], and structurally equivalent residues were used to calculate the aforementioned variables.

The following experimental structures were used for this analysis:

**NMR structures:** 1ab2, 1aot, 1aou, 1bfi, 1bfj, 1blj, 1blk, 1csy, 1csz, 1fhs, 1fu5, 1fu6, 1ghu, 1hcs, 1hct, 1ju5, 1ka6, 1ka7, 1lui, 1luk, 1lum, 1lun, 1mw4, 1oo3, 1oo4, 1pic, 1qg1, 1rja, 1tce, 1wqu, 1x0n, 1x6c, 1z3k, 2bbu, 2cr4, 2crh, 2cs0, 2dcr, 2dly, 2dlz, 2dm0, 2dvj, 2ecd, 2ekx, 2el8, 2eo3, 2eo6, 2eob, 2etz, 2eu0, 2eyv, 2eyy, 2eyz, 2fci, 2ge9, 2gsb, 2jyq, 2k79, 2k7a, 2kk6, 2kno, 2l3t, 2l4k, 2l6k, 2lct, 2lnw, 2lnx, 2lqn, 2lqw, 2mc1, 2mk2, 2mqi, 2mrj, 2mrk, 2pld, 2ple, 2pna, 2pnb, 2rmx, 2ror, 2rsy, 2rvf, 2ysx, 2yu7, 3hck.

**X-ray structures:** 1a07, 1a08, 1a09, 1a1a, 1a1b, 1a1c, 1a1e, 1a81, 1ad5, 1aya, 1ayb, 1ayc, 1ayd, 1bf5, 1bg1, 1bhf, 1bhh, 1bkl, 1bkm, 1bm2, 1bmb, 1cj1, 1cwd, 1cwe, 1d1z, 1d4t, 1d4w, 1f1w, 1f2f, 1fbz, 1fmk, 1fyr, 1g83, 1gri, 1h9o, 1i3z, 1ijr, 1is0, 1jwo, 1jyq, 1jyr, 1jyu, 1k9a, 1kc2, 1ksw, 1lcj, 1lck, 1lkk, 1lkl, 1m27, 1m61, 1mil, 1nrv, 1nzl, 1nzv, 1o41, 1o42, 1o43, 1o44, 1o45, 1o46, 1o47, 1o48, 1o49, 1o4a, 1o4b, 1o4c, 1o4d, 1o4e, 1o4f, 1o4g, 1o4h, 1o4i, 1o4j, 1o4k, 1o4l, 1o4m, 1o4n, 1o4o, 1o4p, 1o4q, 1o4r, 1opk, 1opl, 1p13, 1qad, 1qcf, 1r1p, 1r1q, 1r1s, 1rpy, 1rqq, 1sha, 1shb, 1shd, 1skj, 1spr, 1sps, 1tze, 1uur, 1uus, 1x27, 1xa6, 1y1u, 1y57, 1yvl, 1zfp, 2abl, 2aoa, 2aob, 2aug, 2b3o, 2c0i, 2c0o, 2c0t, 2c9w, 2ci8, 2ci9,

2cia, 2dx0, 2fo0, 2h46, 2h5k, 2h8h, 2hck, 2hdv, 2hdx, 2hmh, 2huw, 2iug, 2iuh, 2iui, 2izv, 2oq1, 2ozo, 2ptk, 2qms, 2shp, 2src, 2vif, 2y3a, 3bkb, 3c7i, 3cbl, 3cd3, 3cwg, 3cxl, 3eac, 3eaz, 3gqi, 3gxw, 3gxx, 3hhm, 3hiz, 3imd, 3imj, 3in7, 3in8, 3k2m, 3kfj, 3m7f, 3maz, 3mxc, 3mxy, 3n7y, 3n84, 3n8m, 3nhn, 3ov1, 3ove, 3pjp, 3pqz, 3ps5, 3psj, 3psk, 3qwx, 3qwy, 3s8l, 3s8n, 3s8o, 3s9k, 3t04, 3tkz, 3tl0, 3uf4, 3us4, 3uyo, 3vrn, 3vro, 3vrp, 3vry, 3vrz, 3vs0, 3vs1, 3vs2, 3vs3, 3vs4, 3vs5, 3vs6, 3vs7, 3wa4, 4d8k, 4dgp, 4dgx, 4e68, 4e93, 4eih, 4ey0, 4f59, 4f5a, 4f5b, 4fbn, 4fl2, 4fl3, 4gl9, 4gwf, 4h1o, 4h34, 4je4, 4jeg, 4jgh, 4jmg, 4jmh, 4jps, 4k11, 4k2r, 4k44, 4k45, 4l1b, 4l23, 4l2y, 4lud, 4lue, 4m4z, 4nwf, 4nwg, 4ohd, 4ohe, 4ohh, 4ohi, 4ohl, 4ovu, 4ovv, 4p9v, 4p9z, 4qsy, 4roj, 4tzi, 4u17, 4u1p, 4u5w, 4waf, 4wwq, 4x6s, 4xey, 4xi2, 4xz0, 4xz1, 4y5u, 4y5w, 4ykn, 4z32, 4zop, 5aul, 5bo4, 5cdw, 5d0j, 5d39, 5dc0, 5dc4, 5dc9, 5df6, 5eel, 5eeq, 5eg3, 5ehp, 5ehr, 5fi4, 5i6v, 5ibm, 5ibs, 5itd, 6cmp, 6cmr, 6cms, 6crg

**Table S1. Simulations of complexes created starting from X-ray structures)**

The simulated sequence (2nd column) was modeled from the original sequence (3rd column) as present in the respective PDB structure (PDB code in 4th column). Substitutions (red characters), were performed with Molecular Operative Environment (MOE), followed by conformational analysis, local energy minimization with side chain repacking. Residues in lowercase were not resolved in the crystallographic structures. Italics indicates residues that were deleted from the original sequence. Underlining indicates residues that were added, with the same procedure.

| ID | Sequence | Original Sequence | PDB.chain |
|---|---|---|---|
| **GAB1_10** | QVE-pY-LDLDLD | gd*K*QVE-pY-LDLDLD | 4QSY.B |
| **GAB1_13** | GDKQVE-pY-LDLDLD | gdKQVE-pY-LDLDLD | 4QSY.B |
| **IRS1-1172_8** | LN-pY-IDLDL | gd*K*QVE-pY-LDLDL*D* | 4QSY.B |
| **IRS1-1171_9** | LN-pY-IDLDLV | gd*K*QVEpYLDLDLD | 4QSY.B |
| **IRS1-1172_11** | SLN-pY-IDLDLVK | gd*K*QVEpYLDLDLD | 4QSY.B |
| **IRS1-1172_12** | SLN-pY-IDLDLVKD | gd*K*QVE-pY-LDLDLD | 4QSY.B |
| **IRS1-895** | PGE-pY-VNIEFGS | spGE-pY-VNIEFgs | 1AYB.P |
| **IMHOF9** | AALN-pY-AQLMFP | SVL-pY-TAVQPne | 1AYA.P |
| **SWEENEY12** | VL-pY-MQPLNGRK | *S*VL-pY-TAVQPne | 1AYA.P |
| **IRS1-546** | IEE-pY-TEMMPAA | SVL-pY-TAVQPne | 1AYA.P |
| **PDGFR-1009** | SVL-pY-TAVQPNE | SVL-pY-TAVQPne | 1AYA.P |
| **IMHOF5** | RLN-pY-AQLWHR | rLN-pY-AQLWhr | 3TL0.B |

Table S2. Solvent Exposure of Phosphopeptide Residues

| | ID | −6 | −5 | −4 | −3 | −2 | −1 | 0 | +1 | +2 | +3 | +4 | +5 | +6 | +7 | +8 |
|---|---|---|---|---|---|---|---|---|---|---|---|---|---|---|---|---|
| PDB | 4QSY | | | K | Q | V | E | pY | L | D | L | D | L | D | | |
| | | | | 60 | 61 | 14 | 53 | | 5 | 62 | 7 | 51 | 7 | 42 | | |
| | 1AYB | | | | | G | E | pY | V | N | I | E | F | | | |
| | | | | | | - | 46 | | 10 | 56 | 3 | 56 | 24 | | | |
| | 1AYA | | | | S | V | L | pY | T | A | V | Q | P | | | |
| | | | | | 46 | 6 | 70 | | 22 | 27 | 8 | 33 | 35 | | | |
| | 5X94 | | | | | P | I | pY | A | T | I | D | F | D | | |
| | | | | | | 27 | 43 | | 0 | 50 | 5 | 27 | 21 | 60 | | |
| | 3TL0 | | | | | L | N | pY | A | Q | L | W | | | | |
| | | | | | | 26 | 46 | | 0 | 50 | 5 | 42 | | | | |
| | 5DF6 | | | | | | T | pY | T | E | V | D | | | | |
| | | | | | | | 65 | | 15 | 63 | 1 | 41 | | | | |
| | 5X7B | | | | | P | I | pY | A | T | I | D | | | | |
| | | | | | | 44 | 56 | | 5 | 52 | 18 | 60 | | | | |
| MD | GAB1_10 | | | | Q | V | E | pY | L | D | L | D | L | D | | |
| | | | | | 88 | 98 | 85 | | 45 | 78 | 7 | 74 | 26 | 72 | | |
| | GAB1_13 | G | D | K | Q | V | E | pY | L | D | L | D | L | D | | |
| | | - | 95 | 87 | 98 | 99 | 69 | | 17 | 75 | 14 | 60 | 45 | 83 | | |
| | IRS1-1172_8 | | | | | L | N | pY | I | D | L | D | L | | | |
| | | | | | | 23 | 41 | | 6 | 46 | 9 | 40 | 21 | | | |
| | IRS1-1172_9 | | | | | L | N | pY | I | D | L | D | L | V | | |
| | | | | | | 75 | 74 | | 22 | 77 | 22 | 60 | 24 | 81 | | |
| | IRS1-1172_11 | | | | S | L | N | pY | I | D | L | D | L | V | K | |
| | | | | | 55 | 64 | 94 | | 29 | 75 | 14 | 57 | 68 | 75 | 78 | |
| | IRS1-1172_12 | | | | S | L | N | pY | I | D | L | D | L | V | K | D |
| | | | | | 95 | 31 | 76 | | 9 | 77 | 14 | 62 | 33 | 72 | 86 | 84 |
| | IRS1-895 | | | S | P | G | E | pY | V | N | I | E | F | G | S | |
| | | | | 77 | 92 | - | 97 | | 25 | 78 | 26 | 54 | 36 | - | 87 | |
| | IMHOF9 | | | A | A | L | N | pY | A | Q | L | M | F | P | | |
| | | | | 88 | 96 | 77 | 82 | | 21 | 80 | 17 | 58 | 38 | 74 | | |
| | SWEENEY12 | | | | | V | L | pY | M | Q | P | L | N | G | R | K |
| | | | | | | 24 | 76 | | 8 | 80 | 36 | 46 | 93 | - | 45 | 90 |
| | IRS1-546 | | | | I | E | E | pY | T | E | M | M | P | A | A | |
| | | | | | 91 | 95 | 91 | | 45 | 77 | 8 | 57 | 68 | 20 | 95 | |
| | PDGFR-1009 | | | | S | V | L | pY | T | A | V | Q | P | N | E | |
| | | | | | 90 | 49 | 90 | | 35 | 71 | 12 | 55 | 76 | 37 | 96 | |
| | IMHOF5 | | | | R | L | N | pY | A | Q | L | W | H | R | | |
| | | | | | 87 | 79 | 93 | | 25 | 79 | 17 | 36 | 80 | 92 | | |

Percentage of sidechain solvent exposed surface area. Exposure is colored in green when lower that 50% and in red when higher than 50%. For MD simulations an average value is reported. Hydrophobic, anionic and cationic residues are colored in green, red and blue, respectively.
